# Cabozantinib and dasatinib synergize to induce tumor regression in non-clear cell renal cell carcinoma

**DOI:** 10.1016/j.xcrm.2021.100267

**Published:** 2021-05-07

**Authors:** Hui-wen Lue, Daniel S. Derrick, Soumya Rao, Ahna Van Gaest, Larry Cheng, Jennifer Podolak, Samantha Lawson, Changhui Xue, Devin Garg, Ralph White, Christopher W. Ryan, Justin M. Drake, Anna Ritz, Laura M. Heiser, George V. Thomas

**Affiliations:** 1 Knight Cancer Institute, Oregon Health and Science University, Portland, OR, USA; 2 Department of Biomedical Engineering, Oregon Health and Science University Center for Spatial Systems Biomedicine, Portland, OR, USA; 3 Graduate Program in Quantitative Biomedicine, Rutgers University, Piscataway, NJ, USA; 4 Department of Pharmacology, University of Minnesota, Minneapolis, MN, USA; 5 Division of Hematology and Oncology, Oregon Health and Science University, Portland, OR, USA; 6 Department of Urology, University of Minnesota, Minneapolis, MN, USA; 7 Masonic Cancer Center, University of Minnesota, Minneapolis, MN, USA; 8 Department of Biology, Reed College, Portland, OR, USA; 9 Department of Pathology and Laboratory Medicine, Oregon Health and Science University, Portland, OR, USA

**Keywords:** kidney cancer, non-clear cell renal cell carcinoma, combination therapies, cabozantinib, dasatinib, cobimetinib, VEGFR, SRC, MEK, high throughput screen

## Abstract

The lack of effective treatment options for advanced non-clear cell renal cell carcinoma (NCCRCC) is a critical unmet clinical need. Applying a high-throughput drug screen to multiple human kidney cancer cells, we identify the combination of the VEGFR-MET inhibitor cabozantinib and the SRC inhibitor dasatinib acts synergistically in cells to markedly reduce cell viability. Importantly, the combination is well tolerated and causes tumor regression *in vivo*. Transcriptional and phosphoproteomic profiling reveals that the combination converges to downregulate the MAPK-ERK signaling pathway, a result not predicted by single-agent analysis alone. Correspondingly, the addition of a MEK inhibitor synergizes with either dasatinib or cabozantinib to increase its efficacy. This study, by using approved, clinically relevant drugs, provides the rationale for the design of effective combination treatments in NCCRCC that can be rapidly translated to the clinic.

## Highlights

Non-clear cell renal cell carcinomas have poor prognoses and limited treatment options
Drug screens identify cabozantinib and dasatinib combination as synergistic in NCCRCC
Combining cabozantinib with dasatinib induces regression of NCCRCC tumors in mice
Integrated multi-omic analyses links therapeutic efficacy to suppression of MAPK activity

## Graphical abstract

**Figure:**
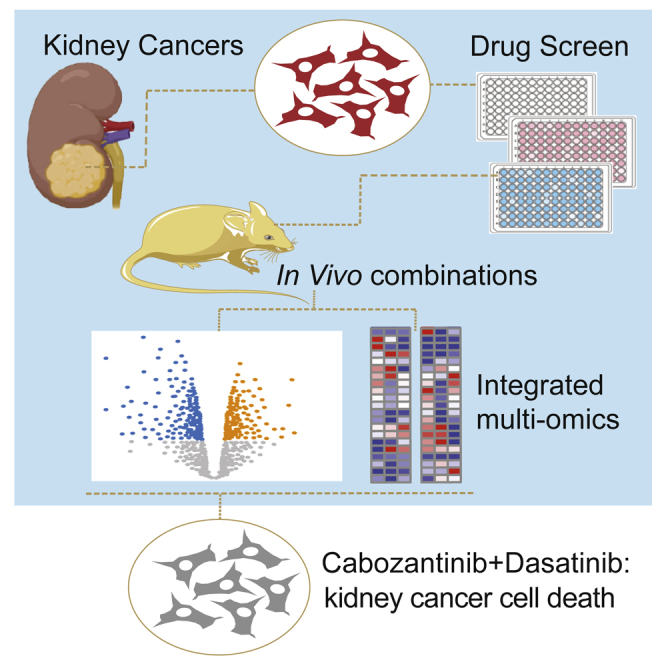

## Teaser

One important and challenging question in kidney cancer is how do we treat treat non-clear cell kidney cancers? Lue et al. tackle this question by performing drug screens in non-clear kidney cancer cells and mouse models and identify the cabozantinib-dasatinib combination as a promising clinically relevant therapeutic option.

## Introduction

Renal cell carcinoma (RCC) accounts for >400,000 new cancer cases and 175,000 deaths per year worldwide.^1^, ^2^, ^3^ Approximately 75% of kidney cancers are predominantly composed of clear cells and characterized by increased angiogenesis due to the loss of function of the *VHL* tumor suppressor gene. Consequently, clear cell RCC (CCRCC) is responsive to drugs that directly or indirectly inhibit angiogenesis.^4^, ^5^, ^6^ The remaining ∼25% of kidney cancers, while pathologically heterogeneous (e.g., papillary, chromophobe, clear cell papillary, collecting duct, medullary, sarcomatoid), have an intact *VHL* gene, and are broadly classified as non-clear cell RCC (NCCRCC) or variant histology RCC. NCCRCC show less responsiveness to antiangiogenics and have no effective treatment options.^7^, ^8^, ^9^, ^10^, ^11^, ^12^, ^13^, ^14^ While recent advances in immunotherapeutic approaches have further improved outcomes for metastatic CCRCC, standard therapies for advanced NCCRCC are lacking and long-term survival is poor.^15^

We and others identified SRC, an intracytoplasmic tyrosine kinase, as a novel therapeutic target in RCC.^16^^,^^17^ Despite its promise, this target has shown minimal efficacy; notably, the multikinase inhibitor dasatinib (which inhibits SRC) is primarily cytostatic and fails to kill RCC cells.^17^ Similarly, the clinical activity of SRC inhibitors in other solid tumors has been modest, with rare durable responses.^18^, ^19^, ^20^, ^21^ The latter observation may be because SRC is activated non-mutationally through its interactions with growth factor receptors, where it acts as a rheostat for multiple signaling pathways that mediate proliferation and survival.^22^ Consequently, upfront combinatorial drug therapies that block SRC and its key signaling partner(s) could be more effective than single-agent dasatinib.

Here, we took a systematic approach toward identifying co-targeting strategies for dasatinib by performing a combination drug screen using a chemogenomic library of mechanistically annotated, clinically relevant approved and investigational drugs that inhibit pathways involved in growth, metabolism, and apoptosis in human RCC cell lines that were *VHL* intact (wild type [WT]) and null, resulting in single-agent and dasatinib combination responses. These studies revealed cabozantinib as a promising drug combination with dasatinib. Cabozantinib inhibits several tyrosine kinases that are biologically relevant in RCC, including VEGFRs, MET, and AXL,^23^ and is approved for use in advanced RCC, having demonstrated improved progression-free survival (PFS) versus standard-of-care sunitinib as a first-line treatment in patients with intermediate- or poor-risk metastatic CCRCC,^24^ and showing significant improvements in PFS, objective response rate (ORR), and overall survival (OS) when compared with everolimus in patients treated with prior anti-angiogenic therapy.^25^^,^^26^ Subsequently, we performed *in vivo* testing in representative *VHL* intact RCC models. Strikingly, while single-agent dasatinib and cabozantinib recapitulated the clinical responses to restrain tumor growth, the combination caused marked tumor regression. Comprehensive integration of transcriptome and phosphoproteomic analysis of the combination therapy revealed rewiring of the kinome, with inhibition of mitogen-activated protein kinase (MAPK) signaling required for cytotoxic synergy. Our studies have identified promising drug combinations that transcend lineage and genetic landscape to induce cytotoxicity, suggesting broad utility across different kidney cancer subgroups.

## Results

### Comprehensive high-throughput drug synergy screen

**Figure 1:**
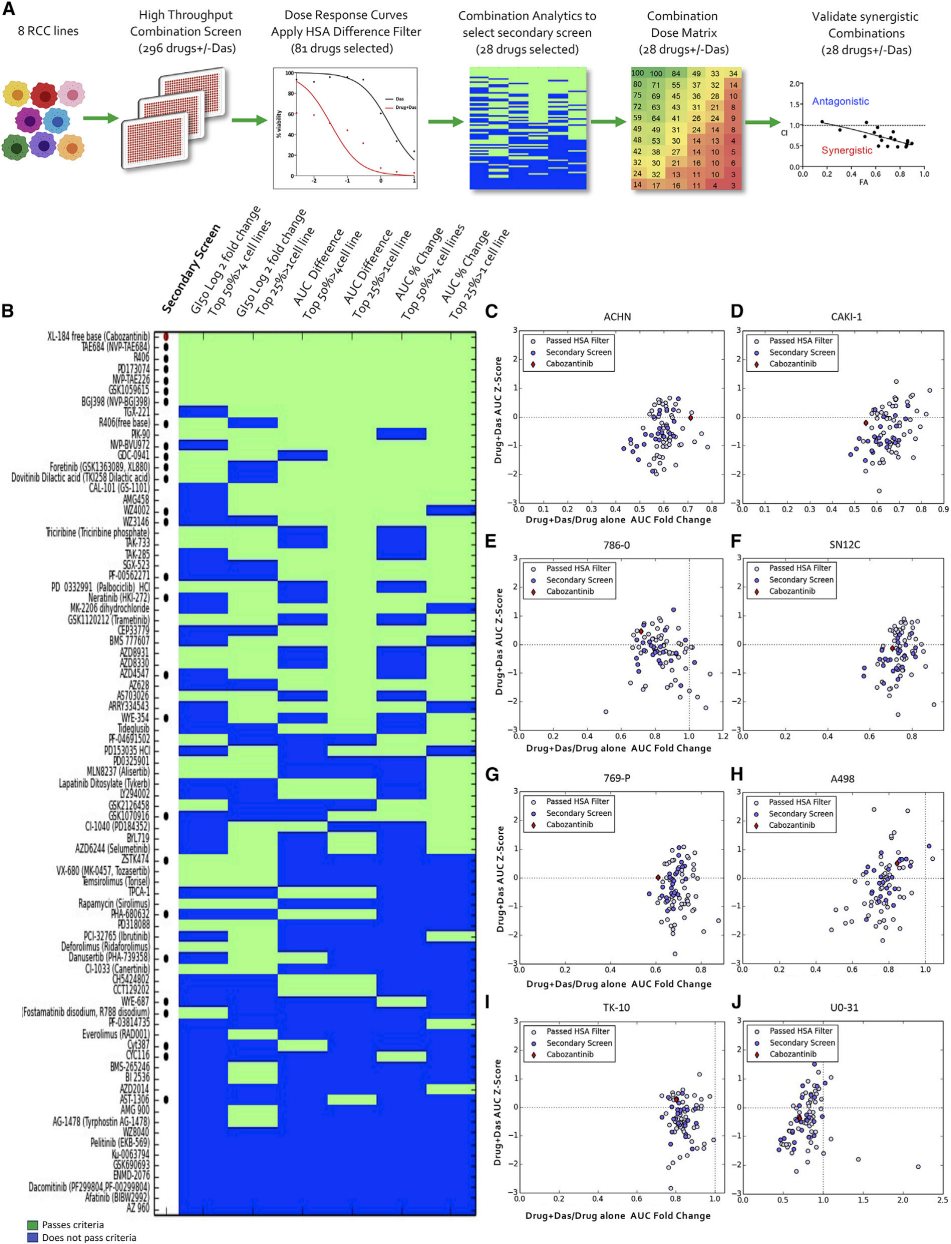
High-throughput drug combination drug screen to identify sensitizers to SRC inhibition in human kidney cancer cells (A) Schematic of the screen workflow: details of the primary screen with 292 drugs ± dasatinib in 8 cell lines are in Figure S1 and Table S1. See Results for additional details. (B) Heatmap of the combination screen of 81 drugs depicting the relative sensitivity of human kidney cancer cells (n = 8: *VHL* wild type: ACHN, SN12C, TK-10, UO-31, CAKI-1; *VHL* Null: 786-0, 769P, A498). The drugs shown passed the “highest single agent” (HSA) filter, where the combination needs to have at least 10% greater inhibition than either dasatinib or the drug alone at the same dose, for at least 3 doses. Each row depicts the response of a drug according to 3 different measurements: G150 fold change (columns 1 and 2), AUC difference between drug alone and drug+dasatinib (columns 3 and 4), and AUC percent change between drug alone and drug+dasatinib (columns 5 and 6). For each criterion, drugs pass (green) or do not pass (blue) 2 thresholds. In the first threshold, the measurement of the drug appears in the top 50% of all measurements in >4 cell lines (odd columns). In the second threshold, the drug measurement appears in the top 25% of all measurements in >1 cell line (even columns). Drugs selected for the secondary screen are denoted with a dot, with cabozantinib labeled with a red dot. (C–J) Scatterplots denote the fold change of drug+dasatinib AUC to drug alone AUC (x axis) versus the drug+dasatinib AUC *Z* score (y axis) for every drug. The red diamond indicates cabozantinib; the dark blue dots indicate drugs selected for the secondary screen; the remaining dots indicate the drugs that pass the HSA filter but were not in the secondary screen. Horizontal dashed line indicates the mean AUC *Z* score of 0, and the vertical dashed line indicates the AUC fold change of 1 (denoting that the drug+dasatinib AUC and the drug alone AUC are the same); (C) ACHN, (D) CAKI-1, (E) 786-0, (F) SN12C, (G) 769-P, (H) A498, (I) TK10, and (J) UO-31.

Hypothesizing that the purely cytostatic response observed with SRC inhibition alone necessitates co-targeting of bypass signaling pathways, we performed a combination drug screen to identify drugs that synergized with dasatinib to kill cancer cells (Figure 1A; Table S1). We screened a library of 292 structurally diverse, medicinally active, and cell-permeable small molecules (including inhibitors of key cancer-relevant targets—for example, VEGFR, MET, EGFR, PDGFR, PI3K, CDK, and apoptosis-inducing molecules such as BCL2, TP53, MDM2, survivin) in 8 human RCC cell lines (*VHL* WT: ACHN, SN12C, TK10, UO31, CAKI-1, and *VHL* Null: 786-0, 769-P, A498; Table S1 [note: CAKI-1 has clear cell pathology]).^27^ We tested 8 doses of each drug with or without dasatinib and read viability after 5 days of drug treatment, thereby generating 37,888 single-agent and drug+dasatinib dose responses. Eighty-one of the drugs passed the “highest single agent” (HSA) filter, in which the combination has at least 10% greater inhibition than either dasatinib or the single agent alone at the same dose, for at least 3 doses.^28^ The viability readings were used to calculate the GI_50_ (the drug concentration necessary to inhibit growth by 50% compared to the untreated condition), the minimum viability, and the AUC (area under the dose-response curve) between drug alone and drug+dasatinib. Subsequently, we determined the leads for secondary screening with the following rationale: a drug should have an effect in multiple cell lines, but an effect across all cell lines is not necessary. Therefore, for a specific measurement such as GI_50_ or AUC, we considered drugs that were in the top 50% of the measurement for all drugs in more than half of the cell lines (i.e., ≥5 cell lines) and drugs that were in the top 25% of the measurement for all drugs in >1 cell line (i.e., ≥2 cell lines). We applied these criteria to 3 calculations (GI_50_ fold change, AUC difference between drug alone and drug+dasatinib, and AUC percent change between drug alone and drug+dasatinib) and noted the drugs that passed the criteria for multiple measurements (Figure S1). In addition, we confirmed the cytostatic effect of single-agent dasatinib in all 8 cell lines (Figure S2A). We shortlisted the drugs based on the above screen criteria for efficacy, safety considerations, and clinical utility and nominated 28 drugs. Notably, these drugs were active against targets relevant to RCC biology, including CDK, mTOR, PI3K, MET and VEGFR. In a composite analysis of all cell lines, cabozantinib emerged as the strongest sensitizer across all parameters (Figure 1B; see STAR Methods). These 28 drugs showed strong curve shifts in combination relative to single agents (drug+dasatinib/drug alone log fold change <1; Figures 1C–1J).

**Figure 2:**
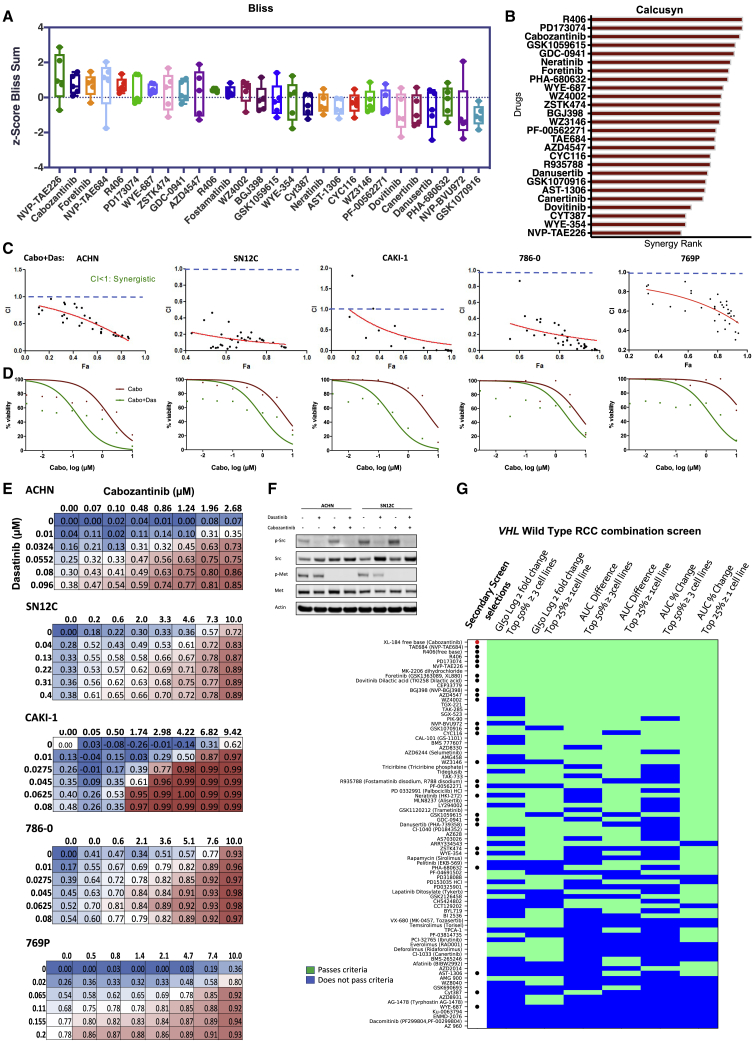
Validation of cabozantinib-dasatinib combination across representative RCC cells (A–C) Dose matrices for 5 human RCC cell lines, ACHN, CAKI-1, SN12C, 786-0, and 769-P, were generated in a 6 × 8 format (6 doses of dasatinib and 8 doses of the drug), assessed for viability after 4 days of treatment, and subjected to the estimation of synergy using the Bliss Independence Model and CalcuSyn. (A) Positive Bliss scores indicate combination effects, in which the effect is greater than additive. (B) CalcuSyn calculates the combination index (CI) for drug combinations: CI < 1 is synergistic, CI = 1 additive, and CI > 1 is antagonistic effects. The response of each cell line to the combination was analyzed for synergy and ranked by the number of combinations that were synergistic (see Method details). (C) CI for the cabozantinib+dasatinib combination in ACHN, SN12C, CAKI-1, 786-0, and 769P cells. (D) Cell viability was assessed by CellTiter-Glo in ACHN, SN12C, CAKI-1, 786-0, and 769P human kidney cancer cells treated with escalating doses of cabozantinib alone (red line) or cabozantinib and a fixed dose of dasatinib at its IC_25_ for ACHN, SN12C, CAKI-1, 786-0, and 789P (green line). The best-fit line represents the variable slope (log(inhibitor) versus response). (E) Secondary screening dose matrix of cabozantinib and dasatinib in ACHN, SN12C, CAKI-1, 786-0, and 769P human kidney cancer cells. Viability was assessed after 4 days. Percent inhibition at each dose of the drug is presented. (F) ACHN and SN12C human kidney cancer cells were seeded and treated with either dasatinib (50 nM) or cabozantinib (10 μM), either alone or in combination. Lysates were made after 24 h of treatment and probed with the indicated antibodies. (G) Heatmap of the combination screen of 81 drugs depicting the relative sensitivity of *VHL* WT human kidney cancer cells (n = 5; ACHN, SN12C, TK-10, UO-31, and CAKI-1).

Subsequently, the 28 selected drugs were further subjected to secondary screening, which involved a dose matrix of 6 × 8 in 5 human RCC cell lines (*VHL* WT: ACHN, CAKI-1, SN12C; *VHL* Null: 786-0 and 769-P), generating a further 6,720 dose-response signatures. The growth inhibition values from this secondary screen across different drug doses and combinations were analyzed for synergy using the Bliss independence model.^28^ Positive Bliss scores indicate combination effects where the effect is greater than additive. Cabozantinib was identified as one of the most synergistic combinations with dasatinib (Figure 2A). We additionally analyzed for synergy using the multiple drug dose-median effect model as described by Chou and Talalay^29^ (CalcuSyn 2.0, Biosoft). CalcuSyn calculates the combination index (CI) for drug combinations: CI < 1 is synergistic, CI = 1 is additive, and CI > 1 is antagonistic. We calculated CI for 28 drug combinations in 5 cell lines and ranked the drugs based on their synergistic effects in combination with dasatinib. This demonstrated consistent synergy (CI < 1) between dasatinib and the top-ranked drugs from the primary screen, validating our selection criteria. In particular, we observed synergy between dasatinib and the inhibitors of VEGFR (cabozantinib, PD1703074, foretinib) and PI3K (GSK1059615, and GDC-0941; Figures S2B, S2C, and S3). In agreement with the Bliss model, cabozantinib was one of the highest ranked synergistic combinations (Figures 2B and 2C). Notably, we confirmed our prior finding of synergy between SRC and STAT3 inhibition (CYT387), demonstrating the robustness of our screen.^30^ To confirm the findings of the high-throughput screen, we generated dose-response curves for cabozantinib alone and with the IC_25_ (¼ maximal inhibitory concentration) of dasatinib and observed a leftward shift with corresponding decreases in cell viability (Figures 2D and S4A). Next, we treated RCC cells with increasing doses of cabozantinib and dasatinib alone and in combination and observed a synergistic interaction in suppressing proliferation (Figure 2E). Western blot analysis of ACHN and SN12C cells treated with cabozantinib and dasatinib confirmed the inhibition of their respective targets, as indicated by the de-phosphorylation of MET and SRC (Figure 2F).

When we applied our selection criteria (GI_50_ fold change, AUC difference between drug alone and drug+dasatinib, and AUC percent change between drug alone and drug+dasatinib) to the 5 human *VHL* WT RCCs in our panel, cabozantinib remained as one of the top combination partners for dasatinib (Figure 2G). The emergence of cabozantinib as the lead candidate in these RCC screens may suggest that it is able to transcend lineage and genetic background and offer broad utility across different RCC pathologies.

As our screening data were performed using CellTiter-Glo, which is adenosine triphosphate dependent, we additionally examined the dasatinib-cabozantinib combination using alternative assays measuring caspase activation and resazurin metabolism (CellTiter-Blue). In agreement with the CellTiter-Glo data, combined treatment with dasatinib and cabozantinib increased apoptosis and decreased proliferation in RCC cells in the panel compared to single-agent treatment (Figures S4B and S4C). Dosing for *in vitro* studies was based on prior work from our lab and others and is consistent with reports that single-agent cabozantinib causes minimal apoptosis *in vitro*.^17^^,^^30^, ^31^, ^32^, ^33^, ^34^, ^35^, ^36^

These experiments suggested several potential therapeutic partners for dasatinib. We selected cabozantinib for further evaluation because it has been approved for both first- and second-line treatment in CCRCC,^24^, ^25^, ^26^ is being actively studied in ongoing single and combination clinical trials (NCT03541902, NCT04022343, NCT03937219, and NCT03635892), and its distinction as consistently being a top hit throughout our screens in all of the cell lines tested.

### Dasatinib-cabozantinib co-treatment induces tumor regression in human NCCRCC xenograft models

**Figure 3:**
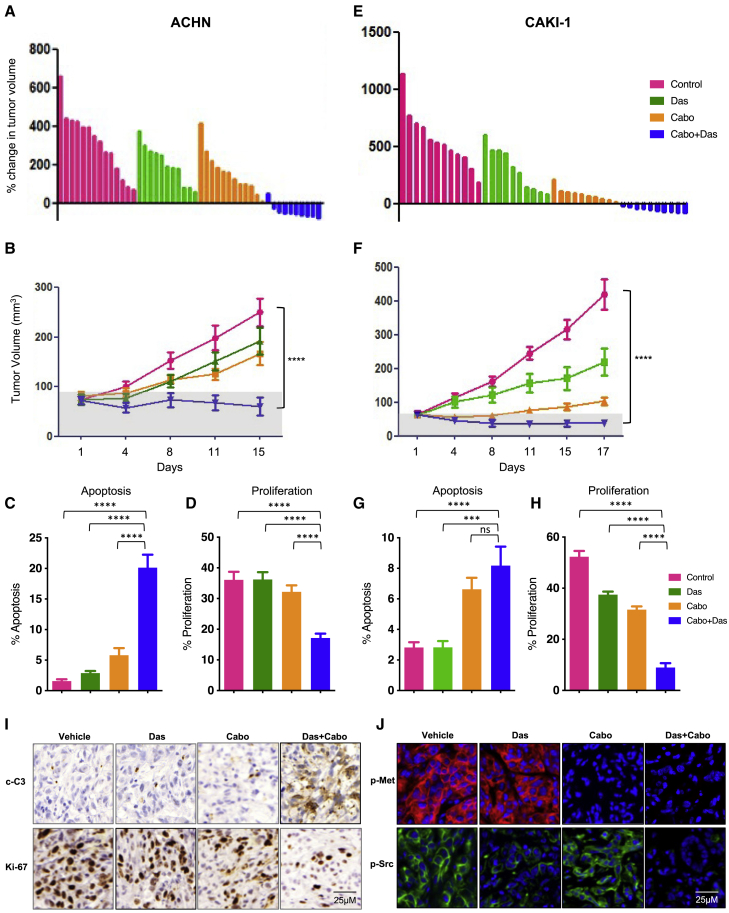
Cabozantinib combines with dasatinib to induce tumor regression (A–D) ACHN xenografts treated with vehicle, dasatinib (das: 25 mg/kg/day), cabozantinib (cabo: 30 mg/kg/day) and dasatinib+cabozantinib (das+cabo: 25 mg/kg/day+30 mg/kg/day) combination. (A) Waterfall representation of response of each tumor after 15 days of treatment is shown. (B) Tumor volume is shown. Error bars represent mean ± SEM; (n > 8 per treatment group; control versus cabo+das ∗∗∗∗p < 0.0001). (C and D) Effect on apoptosis (c-C3) (C) and (D) proliferation (Ki-67) in ACHN xenograft tumors. Error bars represent means ± SEMs (C: control versus cabo+das, ∗∗∗∗p < 0.0001; das versus cabo+das, ∗∗∗∗p < 0.0001; cabo versus cabo+das ∗∗∗∗p < 0.0001). (D: control versus cabo+das, ∗∗∗∗p < 0.0001; das versus cabo+das, ∗∗∗∗p < 0.0001; cabo versus cabo+das ∗∗∗∗p < 0.0001). (E–H) CAKI-1 xenografts treated with vehicle, dasatinib (das: 35 mg/kg/day), cabozantinib (cabo: 10 mg/kg/day), and dasatinib+cabozantinib (das+cabo: 35 mg/kg + 10 mg/kg/day) combination. (E) Waterfall representation of response of each tumor after 15 days of treatment is shown. (F) Tumor volume is shown. Error bars represent means ± SEMs (n > 8 per treatment group; control versus cabo+das ∗∗∗∗p < 0.0001). (G and H) Effect on apoptosis (c-C3) (G) and (H) proliferation (Ki-67) in CAKI-1 xenograft tumors. Error bars represent means ± SEMs (G: control versus cabo+das, p = 0.0002∗∗∗∗; das versus cabo+das, p < 0.0006∗∗∗; cabo versus cabo+das, ns) (H: control versus cabo+das, p < 0.0001∗∗∗∗; das versus cabo+das, p < 0.0001∗∗∗∗; cabo versus*v* cabo+das p < 0.0001 ∗∗∗∗). (I) Representative images of tumor tissue from ACHN xenografts treated with the indicated drug regimens were evaluated by immunohistochemistry for cleaved caspase 3 and Ki-67. (J) Representative images of tumor tissue from ACHN xenografts treated with the indicated drug regimens were evaluated by immunofluorescence for p-MET and p-SRC.

We next examined the safety and efficacy of dasatinib and cabozantinib cotreatment *in vivo* in 2 xenograft tumor models. While dasatinib and cabozantinib exhibited antitumor effects on ACHN and CAKI-1 human RCC xenografts, the combination potently inhibited tumor growth and caused tumor regression (Figures 3A and 3B: ACHN xenograft tumors; Figures 3E and 3F: CAKI-1 xenograft tumors). Importantly, the combination was well tolerated, with no weight loss recorded (Figure S5). Consistent with our prior reports and current *in vitro* findings, dasatinib alone had a minimal impact on apoptosis.^17^^,^^30^ In marked contrast, combination treatment with dasatinib and cabozantinib resulted in a significant increase in the magnitude of apoptosis (established by an increase in cleaved caspase 3; p < 0.0001; ACHN xenograft tumors, CAKI-1 xenograft tumors) and a reduction in proliferation (demonstrated by a decrease in Ki-67; p < 0.0001) (Figures 3C, 3D, and 3I: ACHN xenograft tumors; Figures 3G and 3H: CAKI-1 xenograft tumors). Pharmacodynamic studies demonstrated that combination therapy led to the suppression of SRC and MET-phosphorylation in treated NCCRCC xenograft tumors (Figure 3J). These data support the combination of dasatinib and cabozantinib as an effective strategy for NCCRCC.

### Combination treatment of NCCRCC cells reveals deterministic and stochastic signaling outputs

**Figure 4:**
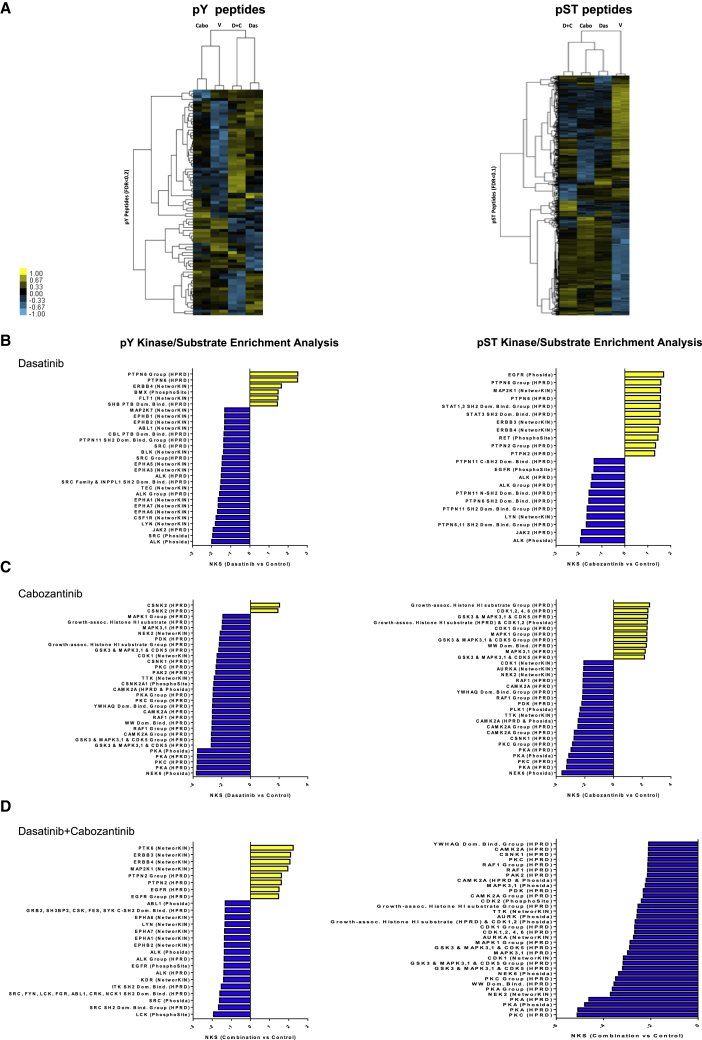
Characterization of the phosphoproteome in cabozantinib-dasatinib co-treated NCCRCC cells (A) Supervised hierarchical clustering heatmaps of phosphotyrosine peptides (pY, left panel), and phosphoserine and phosphothreonine peptides (pST, right) and identified from cabozantinib, dasatinib, and the combination in treated and untreated ACHN human RCC cells with 2 technical replicates. A total of 81 unique pY phosphopeptides (rows) and 3,369 unique pST phosphopeptides were either 4-fold more enriched or 4-fold less enriched, on average (pY: FDR < 0.2; pST: FDR < 0.1; t test p < 0.2), in combination-treated cells compared to untreated cells. (B–D) Kinase-substrate enrichment analysis (KSEA) of (B) dasatinib, (C) cabozantinib, (D) cabozantinib-dasatinib co-treated and untreated pY (hits ≥ 3; FDR < 0.05; left panels), and pST data (hits ≥ 30; FDR < 0.01; right panels). Positive NKS (normalized Kolmogorov-Smirnov score) infers greater kinase activity in cabozantinib-dasatinib co-treated cells, while negative NKS indicates greater activity in untreated cells (unfiltered summary is in Table S2).

To obtain further insight into the signaling pathways acutely affected by dasatinib and cabozantinib cotreatment, we evaluated the changes in the phosphoproteome of the human NCCRCC cell line ACHN after dasatinib, cabozantinib, and the combination treatment via phosphopeptide enrichment coupled to quantitative, label-free quantitative mass spectrometry.^37^, ^38^, ^39^ Supervised hierarchical clustering revealed duplicate samples clustered together, but that treatment altered phosphorylation levels of phosphopeptides, with 3,369 phosphoserine and phosphothreonine (pST) peptides and 81 phosphotyrosine (pY) peptides significantly differed between treated and untreated cells (false discovery rate [FDR] < 0.10–0.20, respectively) (Figure 4A). Next, we applied kinase-substrate enrichment analysis (KSEA),^38^^,^^40^ an approach that estimates changes in the activity of a kinase based on the collective phosphorylation changes of its identified substrates to the single and combination treated cells. KSEA is tailored for phosphoproteomic datasets and is able to predict both known and previously unknown kinase substrates. Several pY kinase substrates were significantly de-enriched after treatment, including SRC, ALK, EPHA3 (dasatinib), NEK6, CAMK2A, and PKC (cabozantinib), and these overlapped in the combination. Conversely, we observed the predicted activity of 3 kinases (PTK6, ERBB, and MAP2K1) via enrichment of their respective pY motifs, all of which may interact with one another, suggesting the potential activation of bypass tracks from combination therapy (Figures 4B–4D and S6; Table S2).

Collectively, the phosphoproteome data provide strong evidence that the combination of cabozantinib and dasatinib affects multiple known and unexpected signaling networks that may be direct or indirect targets of these drugs.

### Integration of transcriptomic and phosphoproteomic datasets reveals coordinated inhibition of the MAPK-ERK pathway

**Figure 5:**
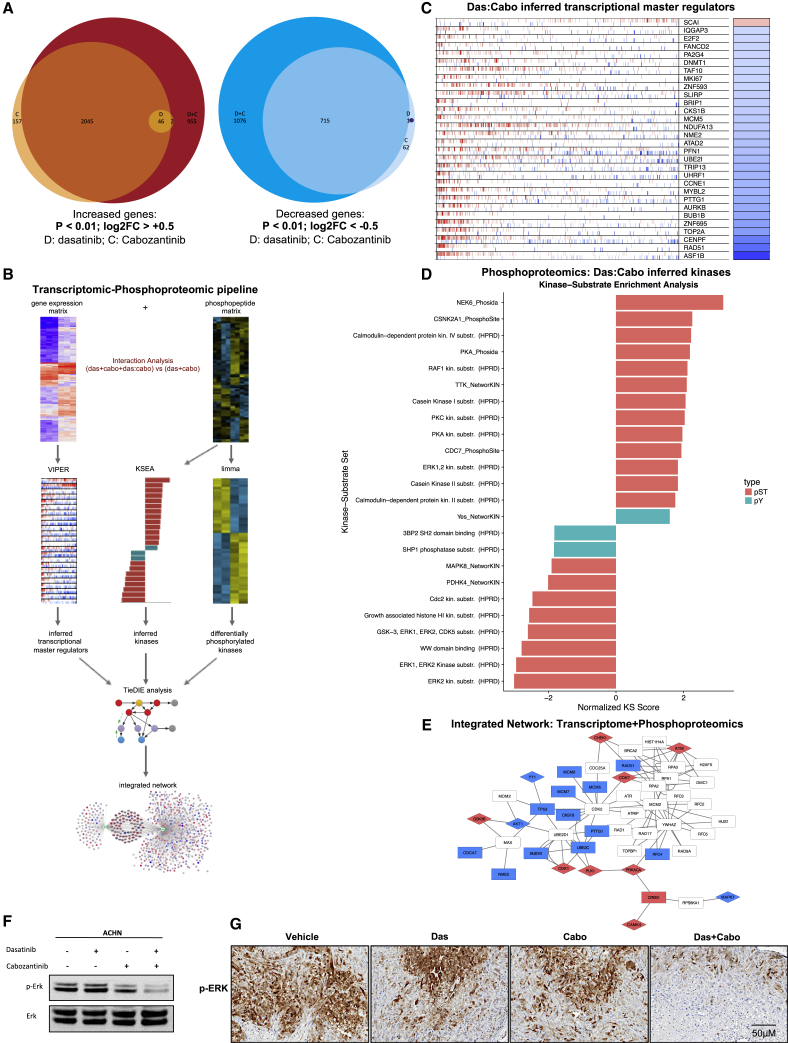
Cabozantinib and dasatinib converge to downregulate the MAPK-ERK signaling pathway (A) RNA: ACHN cells were treated with 50 nM dasatinib (D), 10 μM cabozantinib (C), or the combination (D+C) for 24 h. Differentially expressed genes induced by single or combination drug treatment. Euler diagrams show overlaps in genes with significant increase (log2FC ≥ 0.5; FDR-corrected p ≤ 0.01; left panel) or decrease (log2FC ≤ −0.5; FDR-corrected p ≤ 0.01; right panel) in expression following individual or combination drug treatment. (B) Transcriptomic-phosphoproteomic data integration workflow: to identify genes and phosphopeptides selectively affected by the cabozantinib-dasatinib drug interaction, we compared full models, including terms for the individual drugs and their interaction, to reduced models that only model the individual drug effects. Genes and phosphopeptides were then ranked by the extent to which their expression was better explained by inclusion of an interaction term. Using these ranked lists, we used the VIPER algorithm to infer master regulator activity from transcriptional profiling data, and we used the KSEA algorithm to infer upstream kinase activity from the phosphoproteomic data. The TieDie algorithm was used with the Multinet interaction network to combine inferred transcriptional master regulators, inferred kinases, and directly measured kinases into an integrated network associated with response to the combination drug treatment. (C) Inferred master regulators induced by cabozantinib-dasatinib combination: RNA: transcriptional master regulators driving the unique response to combination treatment were inferred from genes ranked by the interaction coefficient using VIPER. Hashmarks in each row represent the positions of the regulon genes in a list of all of the genes ranked by the interaction coefficient. Red marks indicate positive targets; blue marks indicate negative targets. Heatmap on the right indicates, for each master regulator, the direction of enrichment (blue = negative; red = positive). The top 30 significant master regulators are shown. (D) Inferred kinases induced by cabozantinib-dasatinib treatment: phosphoproteome: kinase activity driving the effect of drug interaction was inferred from the phosphoproteomic data ranked by the interaction coefficient using KSEA (q < 0.05). (E) Integrated transcriptomic/phosphoproteomic cabozantinib-dasatinib interaction network: RNA and phosphoproteome: cabozantinib-dasatinib interaction network generated from VIPER master regulator enrichment scores, KSEA kinase enrichment scores, and kinase using TieDIE algorithm and Multinet interaction network. Red nodes have increased expression due to the interaction effect; blue nodes have decreased expression due to the interaction effect. Rectangles indicate transcription factors and diamonds indicate kinases. (F) ACHN human kidney cancer cells were seeded and treated with either dasatinib (50 nM) or cabozantinib (10 μM), either alone or in combination. Lysates were made after 24 h of treatment and probed with the indicated antibodies. (G) Representative images of tumor tissue from ACHN xenografts treated with the indicated drug regimens were evaluated by immunofluorescence for p-ERK.

Next, we sought mechanistic understanding of the observed tumor regressions after dasatinib-cabozantinib co-treatment by performing RNA sequencing (RNA-seq) of ACHN human NCCRCC cells treated with dasatinib, cabozantinib, and the combination for 24 h. Based on prior work and reports, robust transcriptional signatures emerge ∼24 h after treatment with drugs that affect cell viability such as cell-cycle- and cell death-related signals. These can be captured by RNA-seq profiling, in addition to more specific treatment-related changes, and inform on mechanisms of action.^41^ Cabozantinib treatment caused the differential expression of 4,026 (2,248 up + 1,178 down) genes compared to vehicle treatment. Dasatinib treatment resulted in a much more modest transcriptional response relative to cabozantinib treatment, inducing the differential expression of 49 (48 up + 1 down) genes compared to vehicle treatment. Cotreatment induced the greatest transcriptional response, resulting in differential expression of 5,839 (3,048 + 2,791) genes compared to vehicle (log2FC ≥ 0.5 or ≤0.5; q < 0.01), 65% of which were shared with either or both of the individual treatments (Figure 5A). Comparison of overall expression profiles across the treatments revealed a high degree of correlation across treatments (Figures S7A and S7B; STAR Methods). These results indicate shared molecular programs between each treatment, but also point to molecular changes specifically induced by the combination treatment.

To home in on the genes and phosphopeptides changes specifically induced by the combination treatment, we developed a statistical and network approach to compare across these treatments (Figure 5B). We analyzed the datasets independently, using likelihood ratio tests to compare “full” linear models, including terms for the effects of dasatinib, cabozantinib, and their interaction, to “reduced” models, which only included terms for cabozantinib and dasatinib individually. We prioritized genes and phosphopeptides that were significantly better explained by the full model.

Within the transcriptomic data, 324 genes were significantly better explained after the inclusion of an interaction term, indicating that these genes are preferentially activated after combination treatment (likelihood ratio test: q < 0.01). To identify molecular nodes that may mediate the response to the combination treatment, we performed a master regulator (MR) analysis to identify transcription factors or signaling molecules that serve as signal integration hubs. Specifically, we used the MARINa (master regulator inference algorithm) and VIPER (virtual proteomics by enriched regulon analysis) algorithms and a gene regulatory network derived from The Cancer Genome Atlas cervical kidney renal papillary cell carcinoma (TCGA KIRP) cohort to infer MR activity from genes ranked by the effect of interaction of expression^42^^,^^43^ (Figure 5C). The dasatinib-cabozantinib interaction inferred that transcriptional MRs (e.g., ASF1B, RAD51, CENF, TOP2A, BUB1B, AURKB, PTTG1, MCM5, CCNE1) were involved in DNA replication and repair, transcriptional regulation, proliferation, and mitosis.^44^^,^^45^ Accordingly, gene set enrichment analysis (GSEA) of the inferred transcriptional MRs demonstrated downregulation for cell cycle, DNA replication, homologous recombination, and base excision repair^46^ (Figure S7C).

We used a similar analytical approach to analyze the proteomic data and identified 959 proteins that could be explained by an interaction between dasatinib and cabozantinib. Kinase activity driving the effect of drug interaction was inferred from the phosphoproteomic data using KSEA (q < 0.01), and demonstrated the concordant downregulation of cell-cycle-associated kinases CDK1/CCD2. Notably, KSEA also suggested the downregulation of multiple nodes of the MAPK pathway (e.g., SHP, ERK1, ERK2, ERK5 [MAPK7], JNK1 [MAPK8]) with the dasatinib-cabozantinib combination^47^ (Figure 5D).

To integrate across transcriptional and phosphoproteomic datasets, we leveraged the TieDIE algorithm with the Multinet interaction network to combine inferred transcriptional MRs, inferred kinases, and kinases into an integrated network associated with response to the combination drug treatment.^48^ TieDIE analysis predicted a complex regulatory network underlying the response to the cabozantinib-dasatinib combination treatment that converged to downregulate the MAPK signaling pathway. Specifically, it connected downstream nuclear translocating proteins of the MAPK pathway such as MAPK7 (ERK5), RPS6KA1 (P90-RSK1) to the inferred transcriptional MRs involved in DNA replication and proliferation (e.g., BUB1B, MCM5, PTTG1; Figure 5E).^49^ Critically, in support of the TieDIE “interactome,” western blot analysis of ACHN cells treated with dasatinib or cabozantinib singly demonstrated only minimal effects on MAPK activity (as determined by ERK1/2 phosphorylation), while the combination markedly suppressed MAPK activity (Figure 5F). In addition, the combination therapy led to the suppression of ERK-phosphorylation in treated NCCRCC xenograft tumors (Figure 5G).

**Figure 6:**
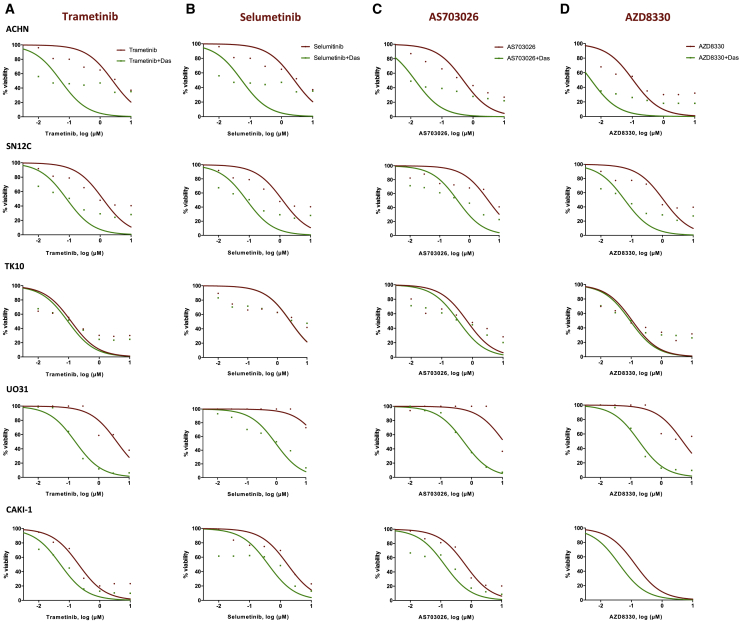
The validation of combining dasatinib and active clinical MEK inhibitors across *VHL* WT human kidney cancer cells (A–D) Dose response to MEK inhibitors is presented both as single agent (red) and in the presence of dasatinib (green). The best-fit line represents the variable slope (log(inhibitor) versus response). (A) trametinib; (B) selumetinib; (C) AS703026; and (D) AZD8330 (n = 4 inhibitors).

**Figure 7:**
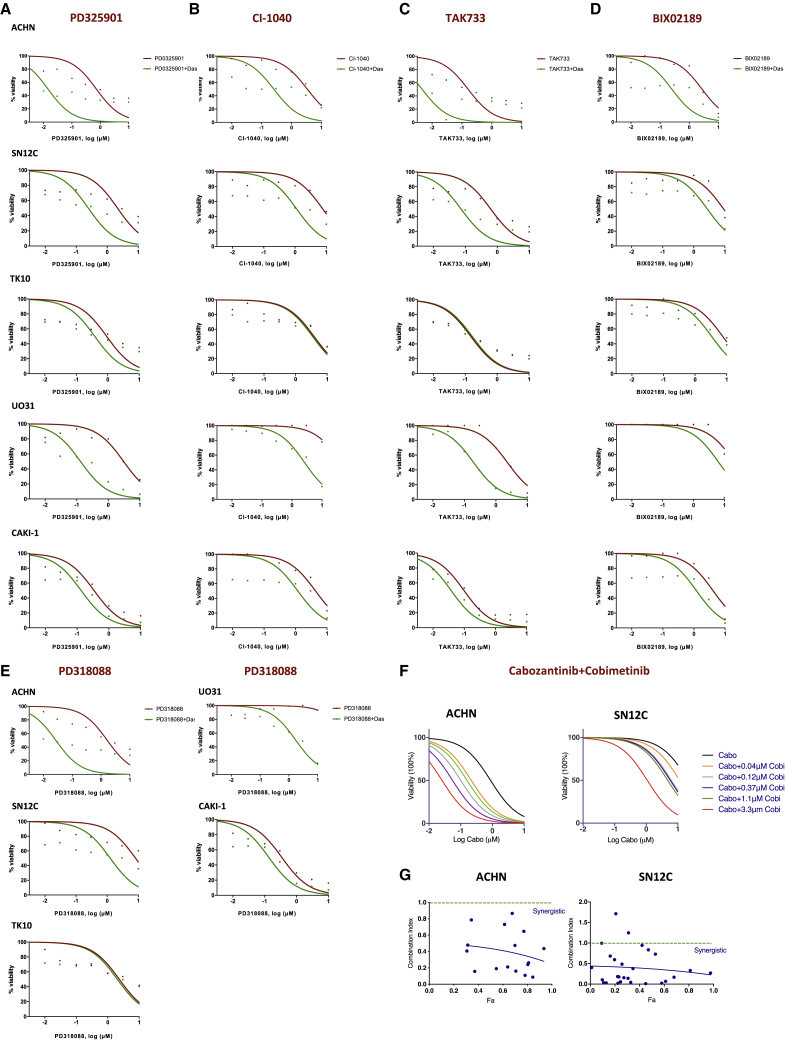
Combination assessments of dasatinib and preclinical MEK inhibitors, and the effect of the cabozantinib-cobimetinib combination (A–E): Dose response to MEK inhibitors is presented both as single agent (red) and in the presence of dasatinib (green). The best-fit line represents the variable slope (log(inhibitor) versus response). (A) PD325901, (B) CI-1040, (C) TAK733, (D) BIX02189, and (E) PD318088 (n = 5 inhibitors). (F) Dose-response curves of cell viability of human NCCRCC cell lines ACHN and SN12C to varying doses of cabozantinib and cobimetinib after 72 h of exposure. (G) Calculated median effect drug synergy CI scores (CalcuSyn) across cabozantinib and cobimetinib combinations. Horizontal dashed line indicates a CI = 1, where points below the line indicate synergy and points above indicate antagonism.

To better define the effect of MAPK pathway inhibition, we examined 9 drugs with known MEK inhibitor activity (trametinib, selumetinib, AS703026, AZD8330, PD325901, CI-1040, TAK733, BIX02189, PD318088) in combination with dasatinib against the 5 VHL WT RCC cells (ACHN, SN12C, TK-10, UO-31, and CAKI-1) in our screen. We generated drug dose-response curves for these MEK inhibitors alone and with dasatinib to examine the drug effect. All 9 structurally unrelated MEK inhibitors have limited single-agent activity, but they were more potent in combination with dasatinib, as seen by the decreased cell viability and leftward shift in dose-response curves (Figures 6 and 7A–7E). Interestingly, TK10 cells consistently demonstrated little or no increase in sensitivity with the addition of dasatinib and may explain why MEK inhibitors were not ranked higher in our screen. Next, we also determined whether the combination of cabozantinib with MEK inhibitor may similarly shift the dose-response curves. To that end, we evaluated the combination of an approved MEK inhibitor undergoing clinical trials in kidney cancer (cobimetinib^50^: NCT03264066) with cabozantinib in 2 VHL WT NCCRCC cells (ACHN and SN12C). In both cancer cell lines, the cobimetinib-cabozantinib combination exhibited marked decreases in cell viability and were synergistic (CI < 1) (Figures 7F and 7G). These results were additionally validated in TK10 and CAKI-1 NCCRCC cells (Figure S8A). These results suggest that the dasatinib-cabozantinib combination may decrease cell viability through the coordinate suppression of MAPK signaling.

We next applied our transcriptional signature to the LINCS (Library of Integrated Network-based Cellular Signature) resource, L1000FWD, which provides interactive visualization of 16,000 drug and small-molecule-induced gene expression signatures in 68 cell lines.^51^ Mechanism of action enrichment analysis revealed MEK inhibitors to be significantly enriched in this dataset, including for selumetinib, which is in our screen (p = 1.78 × 10^−20^). Conversely, antimetabolite chemotherapy agents were significantly de-enriched by the same analysis—for example, gemcitabine (p = 4.52 ×10^−22^) (Figure S8B). These suggest that our drug combinations and mechanistic insights may be applicable to other cancer cell lines

## Discussion

NCCRCC (or variant histology RCC) accounts for ∼25% of kidney cancer, with papillary, chromophobe, clear cell papillary, collecting duct, medullary, and sarcomatoid variants accounting for the majority of subtypes.^52^ Each subtype is likely driven by unique genomic alterations, some of which are better understood (e.g., subsets of papillary RCCs that are driven by the *MET* oncogene, chromophobe tumors exhibit alterations in mitochondrial DNA, RCCs that occur due to *TFE* translocation) and some that are not. For advanced NCCRCC, the only treatment options have been largely extrapolated from agents studied in CCRCC, including anti-angiogenics and mTOR inhibitors. In general, studies have demonstrated that patients with NCCRCC have a worse prognosis, with lower response rates to these therapies.^7^, ^8^, ^9^, ^10^, ^11^^,^^13^^,^^14^^,^^53^^,^^54^ Consequently, the National Comprehensive Cancer Network recommends as the preferred choice enrollment in a clinical trial for NCCRCC patients.^55^

The lack of treatment options for NCCRCC patients represents a critical unmet clinical need. Here, we sought to identify preclinical synergistic drug combinations that would transcend lineage and genetic landscape to induce cytotoxicity in NCCRCC and ultimately lead to deep and durable responses in patients. Accordingly, we present a model to predict and develop novel combination therapies through joint analysis of high-throughput datasets that interrogate multiple aspects of the kidney cancer cell (i.e., drug responses, phosphoproteomics, and transcriptomics). We posit that our phosphoproteomics signaling output likely reflects the direct results of treatment (i.e., primary event), whereas the transcriptional signatures likely represent the consequences that come from signaling pathway modulation (i.e., secondary event). Consequently, integrating the phosphoproteome and transcriptome is a strength because we gained insight into several previously undescribed combinations in NCCRCC—for example, dasatinib with cabozantinib, dasatinib with MEK inhibitors, and cabozantinib with MEK.

We demonstrate that the dasatinib-cabozantinib combination has potent synergy in 2-dimensional (2D) cell culture models. Importantly, this combination induces tumor regression *in vivo*, confirming the robustness of our experimental screening approach. We observed that multiple NCCRCC cells appear to respond similarly to the combination, suggesting broad utility across this histologically diverse group of cancers. Mechanistically, the dasatinib-cabozantinib combination converges to suppress MAPK signaling to induce cytotoxicity. Critically, this suggests that combining dasatinib or cabozantinib with MEK inhibitors could be an alternate combination. In support of this, we observed decreased cell viability with these previously untested combinations. Taken together, our preclinical findings provide rationale for novel combination clinical trial designs.

What then is the most straightforward path to the clinic? We speculate that these findings have direct clinical implications for cabozantinib, providing rationale for further clinical study of cabozantinib in NCCRCC and supporting future combination treatments. Retrospective studies suggest that cabozantinib has activity in patients with NCCRCC^56^^,^^57^ and activity in metastatic RCC to bone.^58^ Results from the randomized Phase II SWOG 1500 study (NCT02761057) demonstrated that cabozantinib treatment significantly increased PFS when compared to sunitinib in patients with metastatic papillary RCC (9 months with cabozantinib versus 5.6 months with sunitinib; hazard ratio 0.60; 95% confidence interval 0.37–0.97; 1-sided p = 0.019).^59^ However, it is highly likely that NCCRCC patients will develop resistance to single-agent cabozantinib, either due to short-term signaling adaptations (e.g., bypass tracks) or longer-term selection of resistance variants (e.g., gatekeeper mutations). Combination studies with cabozantinib need to be considered, and our data suggest that either dasatinib or a MEK inhibitor would be suitable (e.g., cobimetinib, since it is undergoing combination studies in RCC [+azetolizumab, NCT03264066]). Importantly, our work provides the mechanistic rationale on how best to develop these combinations for the clinic.

### Limitations of study

The caveats here are limitations of cell culture and mouse xenograft studies and how these can be extrapolated to clinical trials. While we noted that increasing doses of cabozantinib alone suppressed proliferation in human RCC cells *in vitro*, we and others^32^^,^^34^ only observed the induction of apoptosis by single-agent cabozantinib at higher micromolar doses. In contrast, we observed the robust induction of apoptosis by cabozantinib *in vivo*. This suggests that the cabozantinib may not directly induce apoptosis or may stimulate other types of programmed cell death mechanisms *in vitro*.

Although the dasatinib-cabozantinib combination was well tolerated in mice, the potential clinical toxicities in the setting of patients who have exhausted multiple lines of therapies and with comorbidities are part of the real-world study considerations. Accordingly, appropriate dose-finding studies will be needed as a first step to explore safety and feasibility, either stand-alone or incorporated into larger efficacy trials.

## STAR★Methods

### Key resources table

**Table:** 

REAGENT or RESOURCE	SOURCE	IDENTIFIER
**Antibodies**		

p-SRC	Cell Signaling Technologies	2101
SRC	Cell Signaling Technologies	2108
p-Met	Cell Signaling Technologies	3077
Met	Cell Signaling Technologies	8198
c-Caspase 3	Cell Signaling Technologies	9661
Ki-67	Cell Signaling Technologies	9449
p-ERK	Cell Signaling Technologies	4370
ERK	Cell Signaling Technologies	9102
Actin	Sigma	A5441

**Chemicals, peptides, and recombinant proteins**		

Cabozantinib	Selleck Chem	S4001
Cobimetinib	Selleck Chem	S8041
Dasatinib	Selleck Chem	S1021

**Critical commercial Assays**		

CellTiter-Glo luminescent viability assay	Promega	G8090
Caspase-Glo 3/7 assay	Promega	G7570
CellTiter-Blue cell viability assay	Promega	G8080

**Experimental Models: Cell Lines**		

CAKI-1	ATCC	NA
786-0	NCI	NA
SN12C	ATCC	NA
769-P	NCI	NA
A498	NCI	NA
TK-10	ATCC	NA
U0-31	NCI	NA
ACHN	ATCC	NA

**Experimental Models: Organisms/Strains**		

Mice	J:Nu (Jackson Laboratories)	007850

**Software and Algorithms**		

TrimGalore (v0.4.4)	NA	https://github.com/FelixKrueger/TrimGalore
cutadapt (v1.10)	NA	https://cutadapt.readthedocs.io/en/stable/
STAR (v2.4.0.1)	NA	https://github.com/alexdobin/STAR
Complex Heatmap (v1.17.1)	Bioconductor	https://bioconductor.org/packages/release/bioc/html/ComplexHeatmap.html
DESeq2 (v1.18.1)	Bioconductor	https://bioconductor.org/packages/release/bioc/html/DESeq2.html
Gene Set Enrichment Analysis (v3.0)	NA	https://www.gsea-msigdb.org/gsea/index.jsp
viper (v1.12)	Bioconductor	https://www.bioconductor.org/packages/release/bioc/html/viper.html
limma (v3.36.3)	Bioconductor	https://www.bioconductor.org/packages/release/bioc/html/limma.html
TieDIE (v1.0)	NA	https://sysbiowiki.soe.ucsc.edu/tiedie
MaxQuant (v1.5.3.30)	MaxQuant	https://www.maxquant.org/
Cluster (v3.0)	NA	http://bonsai.hgc.jp/∼mdehoon/software/cluster/software.htm
Prism (v8.0)	GraphPad	https://www.graphpad.com/scientific-software/prism/
Image Studio	LI-COR	https://www.licor.com/bio/image-studio/
inForm (v1.4.0)	Perkin Elmer	https://www.akoyabio.com/phenoptics/software/inform-tissue-finder/

**Other**		

RIPA buffer	Sigma	R0278
Phosphatase inhibitor cocktail set II	EMD Millipore	524625
Phosphatase inhibitor cocktail set III	EMD Millipore	524627
BCA assay	Thermo Scientific	23225
NuPAGE Novex Mini Gel system	Invitrogen	NP0321
Matrigel	BD Biosciences	N/A
Mouse IgG blocking serum	Vector Laboratories	N/A
Immpress mouse IgG	Vector Laboratories	MP7400
Immpress rabbit IgG	Vector Laboratories	MP7401
Immpact DAB	Vector Laboratories	SK4105
Normal goat serum	NA	N/A
Anti-rabbit Alexa 488	Molecular Probes	N/A
Mounting media with DAPI	Dako	N/A

### Resource availability

#### Lead contact

Further information and requests for resources and reagents should be directed to and will be fulfilled by the Lead Contact, George V. Thomas (thomasge@ohsu.edu)

#### Materials availability

This study did not generate new unique reagents

#### Data and code availability

All analysis scripts can be found in the following GitHub repository:

https://github.com/danielderrick/thomas_kidney_drug_analysis

The accession numbers for RNA Seq sequence data reported in this paper are deposited in GenBank: GSE171358

### Experimental model and subject details

#### Cell lines

ACHN, A498, 769-P, 786-O, CAKI-1, SN12C, TK10 and UO31 were used in this study and were obtained from the NCI and ATCC. Cell lines were maintained in Dulbecco’s modified Eagle’s medium (DMEM) supplemented with 10% fetal bovine serum at 37°C in a 5% CO_2_ incubator.

#### High throughput drug screening and workflow on the identification of hits

We did screening using a library of 292 small molecules (that included kinase inhibitors and apoptosis inducing molecules; Table S1: Drugs in the screen; Cell lines, VHL status, Pathology, data associated with cell lines) in 8 cell lines (ACHN, A498, 769-P, 786-O, CAKI-1, SN12C, TK10 and UO31). Quality control measures taken include: determination of plating density (500-1000 cells to allow for exponential growth phase), drug concentrations across 10,000-fold dilution range (0.001nM-10uM), technical replicates for DMSO in each plate (with %CV measured), % CV of technical replicates of dasatinib alone in each plate (with % CV measured), and biological replicates for 2 cell lines (ACHN and TK-10: Table S1). We did 8 dilutions of drug with or without dasatinib and read viability using cell titer glo after 5 days of drug treatment. The readings were used to calculate the GI50 (using XLfit), minimum viability, AUC (area under curve), AUC difference between drug alone and drug+das and Z score values. Drug response curves were analyzed using the XLfit (IDBS Ltd.) curve-fitting tool for Microsoft Excel to determine the GI50 (concentration of 50% growth inhibition relative to T = 0 and Ymax values), the gI-maximum (concentration giving maximum growth inhibition), and the Ymin (bottom of the four-parameter curve at gI-maximum). Fold-change (GI50) was determined as follows: (GI50 single agent drug)/(GI50 combination drug). Percent change AUC was calculated as follows: [(AUC single agent drug-AUC combination drug)/(AUC single agent drug)]∗100.

We applied multiple criteria to identify the hits from the primary screening that were additionally validated in the secondary screening. The following are the criteria used: 1) GI50 fold change: Drugs that have at least 5-fold change in GI50 ratio between drug alone and drug+dasatinib (ratio GI50 drug/ GI50 combination) in 50% of cell lines (n = 38). 2) AUC difference: Drugs that have AUC difference of more than 0.9 (average is 0.9) between drug alone and drug+dasatinib in at least 50% of cell lines (n = 31). 3) AUC % change: Drugs that have AUC % change of more than 25% (average is 25%) between drug alone and drug+dasatinib in 66% (4 of 6) cell lines (n = 31). 4) GI50 of combination: Combinations that are in top 50% (based on GI50 z-score calculation) in 50% (3 of 6) cell lines and pass through the minimum viability cutoff of 15% across cell lines that (average min viability value is looked at) (n = 18). 5) AUC of combination: Combinations that are in the top 50% (based on AUC z-score calculation) and in 50% (3 of 6) cell lines and pass through the minimum viability cutoff of 15% across cell lines that (average min viability value is looked at) (n = 13). We shortlisted the drugs based on the above criteria and picked 28 drugs that were identified by at least 4 of the 5 parameters. These 28 drugs were further subjected to the secondary screening, which involved a dose-matrix of 6X8 (6 doses of dasatinib and 8 doses of the drug). The growth inhibition values from the secondary screening were subjected to the estimation of synergy using Calcusyn. Calcusyn calculates Combination Index (CI) for drug combinations: CI < 1 is synergistic; CI = 1 additive and CI > 1 is antagonistic effects.^29^ We calculated CI for 28 drug combinations and ranked the drugs based on its synergistic effect in combination with dasatinib from highest to lowest.

#### *In vivo* xenograft Studies

All animal experiments were approved by the Institutional Animal Care and Use Committee at OHSU (IACUC protocol: TR02_IP00000022). The studies are compliant with all relevant ethical regulations regarding animal research. All mice were housed in the OHSU animal facility approved by AAALAC in accordance with NIH guidelines. The animal facility provides care for a variety of animals and provides services of feeding and watering, cage cleaning and room sanitation, plus observation and health assessment of laboratory animals. All nude mice were housed in sterile conditions and handled in a sterile laminar airflow room by trained staff, supervised by several veterinarians. Six-week old female mice (J: nu; stain 007850 from Jackson Laboratories) were utilized for human renal cell carcinoma xenografts. For both ACHN and CAKI-1 cell lines 2x10^6^ cells were diluted in 50 μl of PBS and 50 μl of Matrigel (BD Biosciences) and were injected subcutaneously into the right and left flank of each mouse (8 mice per treatment arm). Tumors were monitored until they reached an average size of 50-80mm3 (approximately 2 weeks), at which point treatments were begun. dasatinib (25mg/kg/day: ACHN; 35mg/kg/day: CAKI-1) was administered by oral gavage 5 days/week. Cabozantinib (30mg/kg/day: ACHN; 10mg/kg/day: CAKI-1) were administered by oral gavage 3 days/week. The *in vivo* dosing for dasatinib and cabozantinib were chosen based on prior work from our group and others: Dasatinib has been administered as a single agent in the 25-50mg/kg range^30^^,^^60^, ^61^, ^62^; and cabozantinib as single agent in the 30-100mg/kg range.^32^^,^^35^^,^^63^, ^64^, ^65^, ^66^, ^67^, ^68^ For the dasatinib-cabozantinib co-treatment studies *in vivo*, we examined our *in vitro* 6x8 dose matrix dasatinib-cabozantinib combination results, which demonstrated higher doses of cabozantinib were required to decrease ACHN cell viability and so we chose 25mg/kg dasatinib+30mg/kg cabozantinib *in vivo* dose. Conversely, the *in vitro* dasatinib-cabozantinib combination indicated that lower doses of cabozantinib when combined with higher doses of dasatinib elicited decreased CAKI-1 cell viability. Dasatinib and cabozantinib were dissolved in NMP/PEG. Tumors and mouse weights were measured twice weekly. At least 6-8 mice per treatment group were included in the analyses (based on previous studies performed by us and others, as referenced above), and the investigators were not blinded to the identity of the study groups. All mice were euthanized using CO_2_ inhalation followed by cervical dislocation per institutional guidelines at Oregon Health and Science University.

### Method details

#### Cell viability and apoptosis analysis

Cell viability assays were performed by plating cells/well in 96-well plates in triplicate and treating the following day with the indicated agent: dasatinib (dose range of 0–400 nM) and cabozantinib (dose range of 0–10 μM). These doses were based on prior work from our group and others.^17^^,^^30^, ^31^, ^32^, ^33^, ^34^, ^35^, ^36^ The experiment was continued for 3 days and then the cells were treated with CellTiter-Blue (Promega) and incubated for 1 hour. Fluorescence was measured and quantified and photographs were obtained using a Cytation 5 Cell Imaging Reader. The effect of dasatinib, cabozantinib and the dasatinib+cabozantinib combination on cell number was assessed as fold of DMSO-treated control cells. Experimental results are the average of at least three independent experiments. Apoptosis was determined using Caspase 3/7-Glo assay kit (Promega) following the manufacturer’s instructions. Briefly, 4000 cells per well were plated in 96 well plates and cultured for 24h. Cells were treated with dasatinib, cabozantinib and the combination of dasatinib+cabozantinib for 72h, and then 100 μL reagents were added to each well and incubated for 30 min at room temperature (timing of assay was based on growth kinetics of the cells in the presence of single and combination treatment). Caspase 3/7 activity was measured using a luminometer. Luminescence values were normalized by cell numbers. The effect of dasatinib, cabozantinib and the combination of dasatinib+cabozantinib on caspase 3/7 activation was assessed as fold of DMSO-treated control cells.

#### Immunoblotting

Cells were plated in 6 well dishes and treated the following day with the indicated agents. Treatments were for 24 hours, after which cells were washed with ice cold PBS and lysed with RIPA buffer (Sigma). Phosphatase inhibitor cocktail set II and protease inhibitor cocktail set III (EMD Millipore) were added at the time of lysis. Lysates were centrifuged at 15,000 g x 10 min at 4°C. Protein concentrations were calculated based on a BCA assay (Thermo Scientific) generated standard curve. Proteins were resolved using the NuPAGE Novex Mini Gel system on 4% to 12% Bis-Tris Gels (Invitrogen). For western blotting, equal amounts of cell lysates (15-20 μg of protein) were resolved with SDS-PAGE, and transferred to membranes. The membrane was probed with primary antibodies, washed, and then incubated with corresponding fluorescent secondary antibodies and washed. The fluorescent signal was captured using LI-COR (Lincoln, NE) Odyssey Imaging System, and fluorescent intensity was quantified using the Odyssey software where indicated. The following antibodies were used for western blots: p-SRC (Y416), SRC, p-MET (Y1234/1235), p-p44/42 MAPK (Erk1/2) (T202/Y204), ERK and β-actin (AC15). Ki67 and cleaved caspase 3 were used for immunohistochemistry. Dasatinib, cabozantinib and cobimetinib were purchased from Selleck chemicals.

#### Immunohistochemistry

Immunostaining was performed following deparaffinization and rehydration of slides. Antigen retrieval was performed in a pressure cooker using citrate buffer (pH 6.0) for 4 min. Nonspecific binding was blocked using Vector mouse IgG blocking serum 30 min at room temperature. Samples were incubated at room temperature with rabbit monoclonal antibodies cleaved caspase 3, and Ki67. Slides were developed with Vector Immpress rabbit IgG and Vector Immpress mouse IgG (Vector Laboratories) for 30 min at room temperature. Chromogenic detection was performed using Vector Immpact DAB (Vector Laboratories) for 3 min. Slides were counterstained with hematoxylin. A 3DHistech MIDI Scanner (Perkin Elmer) was used to capture whole slide digital images with a 20x objective. Images were converted to into MRXS files and computer graphic analysis was completed using inForm 1.4.0 Advanced Image Analysis Software (Perkin Elmer).

#### Immunofluorescence

H&E slides of formalin fixed, paraffin embedded tissue was used to assess morphological integrity of tumor samples. Once integrity was confirmed, immunofluorescent analysis was performed for p-SRC (Y416), p-MET (Y1234/1235). Four μ sections were cut, de-paraffinized and rehydrated. Antigen retrieval was performed using citrate for 4 min in a pressure cooker. Slides were blocked using 2.5% normal goat serum for 30 min then incubated in primary antibody for 1hr followed by secondary antibody mouse anti-rabbit Alexa 488 (1:1000, Molecular Probes) for 30 min. Slides were rinsed in PBS, air-dried, and coverslipped using Dako mounting media with Dapi.

### Quantification and statistical analysis

#### RNA-seq preprocessing and analysis

RNA was extracted from triplicate 24-hour treated cell lines using the RNeasy extraction kit (QIAGEN), and the quality if the extracted RNA was verified using an Agilent 2100 Bioanalyzer. Unstranded, poly-A-selected RNA-seq libraries were prepared using a TruSeq RNA Library Prep kit (Illumina). Read depth and splice junction details are in Table S1. Single-end 100bp reads were sequenced with an Illumina HiSeq 2500. Reads were trimmed of low-quality bases and adaptor sequences using TrimGalore (v0.4.4) a wrapper for cutadapt (v1.10).^69^ Reads were aligned to the human reference genome (GRCh38) and summarized to gene-level abundances using STAR (v2.4.0.1).^70^ Gene expression heatmaps were created with the R package Complex Heatmap (v1.17.1).^71^

Differentially expressed genes in drug-treated samples were identified with DESeq2 (v1.18.1)^72^ to compare each treatment (dasatinib, cabozantinib, and dasatinib + cabozantinib co-treatment) to the vehicle-treated condition. Genes with an FDR-corrected q-value ≤ 0.01 and a log2FoldChange ≥ 0.5 or ≤ −0.5 were considered differentially expressed.

Significance of the cabozantinib-dasatinib interaction effect on gene expression was assessed using a likelihood ratio test to compare two generalized linear models. Both models feature logarithmic link functions

$${\log\;}_{2}q_{ij}=\underset{r}{\sum }x_{jr}\beta_{ir}$$

where parameter *q*_*ij*_ is proportional to the predicted true concentration of transcripts in sample *j*, *x*_*jr*_ are the design matrix components for sample *j*, and *β*_*ir*_ are the coefficients (log2 fold changes). The full model

$${\log\;}_{2}q_{ij}=x_{jd}\beta_{id}+\;x_{jc}\beta_{ic}+\;x_{jdc}\beta_{idc}$$

contains design matrix components and coefficients for the dasatinib effect (*x*_*jd*_*β*_*id*_), the cabozantinib effect (*x*_*jc*_*β*_*ic*_), and an interaction effect (*x*_*jdc*_*β*_*idc*_). The reduced model

$${\log\;}_{2}q_{ij}=x_{jd}\beta_{id}+\;x_{jc}\beta_{ic}$$

contains terms only for the dasatinib effect (*x*_*jd*_*β*_*id*_) and the cabozantinib effect (*x*_*jc*_*β*_*ic*_).

We considered genes to be affected by the interaction of the drugs if the full model explained the observed effects significantly better than the reduced model (FDR-corrected q-value < 0.01).

#### Gene set enrichment analysis

Enrichment of specific pathways and ontological terms in the gene most affected by the drug interaction was assessed using the Broad Gene Set Enrichment Analysis (GSEA) (v3.0) Java application^46^ and the Broad MSigDB (v6.2) Hallmark gene set collection. Preranked input for GSEA was generated by ranking genes by the log2 fold change of the by the cabozantinib-dasatinib drug interaction coefficient. P values were calculated by gene label permutation and were FDR-corrected for multiple testing. Gene sets with an FDR-corrected q-value < 0.1 were considered significant.

#### Master regulator analysis

Master Regulator analysis was used to infer transcription factors responsible for the observed drug interaction-induced changes in gene expression. The R package VIPER (v1.12)^42^ was used to test for enrichment of Master Regulators in a list of all genes ranked by the coefficient of the interaction effect (*β*_*idc*_), using a network generated from gene expression profiles of the TCGA kidney renal papillary cell carcinoma cohort.^43^ P-values were calculated by gene label permutation and were FDR-corrected for multiple testing. Master Regulators with an FDR-corrected q-value < 0.1 were considered significant.

#### Phosphoproteomic drug interaction analysis

Interaction effects in the phosphoproteomic data were analyzed in R using the limma (3.36.3)^73^ package. Significance of the cabozantinib-dasatinib interaction effect on phosphoprotein levels was assessed using an empirical Bayes method to compare a full model, which models individual drug and drug interaction effects, to a reduced model, which models only the effects of the individual drugs. The drug interaction was considered to have a significant effect on phosphopeptide abundance if the FDR-corrected q-value was < 0.05.

#### Data integration using TieDie

An integrated network of inferred drug interaction effects on transcription and phosphoprotein levels was created using TieDie (v1.0)^48^ software. TieDie was used to map enriched transcription factors inferred from the RNA-seq data, kinases significantly affected by the drug interaction effect, and enriched kinases inferred from the phosphoproteomic data onto nodes of the Multinet reference network derived from protein-protein interaction, phosphorylation, metabolic, and gene regulatory networks. Inferred master regulators (FDR-corrected q-value < 0.05, NES ≥ 5) were used as the gene expression (“downstream”) input nodes. KSEA-inferred kinases (FDR-corrected q-value ≤ 0.05) were used as the kinase (“upstream”) input nodes. Additionally, directly measured kinases significantly affected by the drug interaction (FDR-corrected q-value ≤ 0.5 for pY phosphopeptides; FDR-corrected q-value ≤ 0.01 for pST phosphopeptides) were included as upstream input. A more stringent q-value threshold was used for pST phosphopeptides to reduce the number of input nodes. The input weight of inferred transcription factors and kinases was equal to their enrichment score; the input weight of directly measured kinases affected by drug interaction was equal to the interaction coefficient *β*_*idc*_. A heat diffusion algorithm was then run to model diffusion of “heat” from the input nodes to nearby nodes in the network, and a subnetwork of agreement between the data types was identified by identifying nodes that receive heat from both data sources.

#### L1000FWD analysis

Similar and opposite drug-induced expression signatures were identified from lists of differentially expressed genes using the L1000 Fireworks Display (L1000FWD) web service. Differentially expressed genes were identified by selecting genes significantly better explained using a full model containing an interaction term, compared to a reduced model containing only terms for single agents (as described above in Method details). The 323 genes (33 upregulated and 290 downregulated) significantly (q < 0.01) better explained by the full model were input to the L1000FWD web GUI. The service returns a list of drug signatures derived from the L1000 platform, ranked by their overlap with the input lists. A hypergeometric test is used to test enrichments for significance.

#### Phosphoproteomic screen and analysis

Phosphopeptides were enriched and analyzed by mass spectrometry as previously described in detail.^32^ Briefly, cells were lysed and proteins extracted using a guanidinium-based lysis buffer. Lysates were digested using Lys-C and trypsin. Phosphotyrosine peptides were enriched via immunoprecipitation (4G10 antibody) and titanium dioxide. Phosphoserine/threonine peptides were enriched by strong cation exchange and titanium dioxide. After desalting with C18 columns, the phosphopeptide samples were subjected to liquid chromatography-tandem mass spectrometry (LC/MS-MS with a dual pump nanoRSLC system (Dionex) interfaced with a Q Exactive HF (Thermo Fisher Scientific)). Technical duplicates were run for all samples and MS raw files were analyzed using MaxQuant version 1.5.3.30^74^ and MS/MS fragmentation spectra were searched using Andromeda^75^ against human canonical and isoform sequences in Swiss-Prot (downloaded in August 2017 from https://www.uniprot.org).^76^

#### Mass spectrometry data analysis

Quantitative phosphopeptide data were log10 transformed and missing data were imputed before applying quantile normalization as previously described.^38^ Hierarchical clustering was performed on the Cluster 3.0 program,^77^ using distance that is based on the Pearson correlation and applying pairwise average linkage analysis. Java Treeview was used to visualize clustering results.^78^

#### Kinase substrate enrichment analysis

Kinase substrate enrichment analysis (KSEA) was performed as previously described.^38^^,^^40^ Briefly, the phosphopeptides were rank ordered by fold change, on average, between combination (dasatinib + cabozantinib) treatment versus control and the enrichment score was calculated using the Kolmogorov-Smirnov statistic. Permutation analysis was conducted to calculate statistical significance. The normalized enrichment score was calculated by dividing the enrichment score by the average of the absolute values of all enrichment scores from the permutation analysis (Table S2).

#### Statistical analysis

Statistical significance between two groups was assessed by unpaired Student’s t test. Mouse tumor size was analyzed by 2-way ANOVA with time and drug as factors, using GraphPad Prism. Mouse weight during treatment was analyzed by repeated-measures 2-way ANOVA, with time and drug as factors. All cell culture experiments were per-formed at least two times. Data are presented as mean ± s.e.m., and a *P value* less than 0.05 was considered statistically significant. Further statistical details, including values of n and definitions of what n represents, can be found in the figure legends. Additional statistical analysis specific to phosphoproteomics and RNA Seq are listed above. Immunohistochemistry: *P*-values were calculated using one-way ANOVA, with Bonferroni’s multiple comparison test. *∗ denotes p < 0.05, ∗∗ denotes p < 0.01, and ∗∗∗ denotes p < 0.001.* The plots here were created using R version 3.6.1. Packages needed include ggplot2 and dplyr.

## Supplemental information

Document S1. Figures S1–S8

Table S1. High-throughput screen

Table S2. Phosphoproteomics KSEA

Document S2. Article plus supplemental information
