# Supplementary material for: Cabozantinib and dasatinib synergize to induce tumor regression in non-clear cell renal cell carcinoma
Source: Cell Rep Med. 2021 May 7;2(5):100267. doi: 10.1016/j.xcrm.2021.100267 (PMC8149375; doi:10.1016/j.xcrm.2021.100267)
Supplement: Document S1. Figures S1–S8 [file mmc1.pdf]

Cell Reports Medicine, Volume *2*

Supplemental information

Cabozantinib and dasatinib synergize

to induce tumor regression in

non-clear cell renal cell carcinoma

Hui-wen Lue, Daniel S. Derrick, Soumya Rao, Ahna Van Gaest, Larry Cheng, Jennifer Podolak, Samantha Lawson, Changhui Xue, Devin Garg, Ralph White III, Christopher W. Ryan, Justin M. Drake, Anna Ritz, Laura M. Heiser, and George V. Thomas

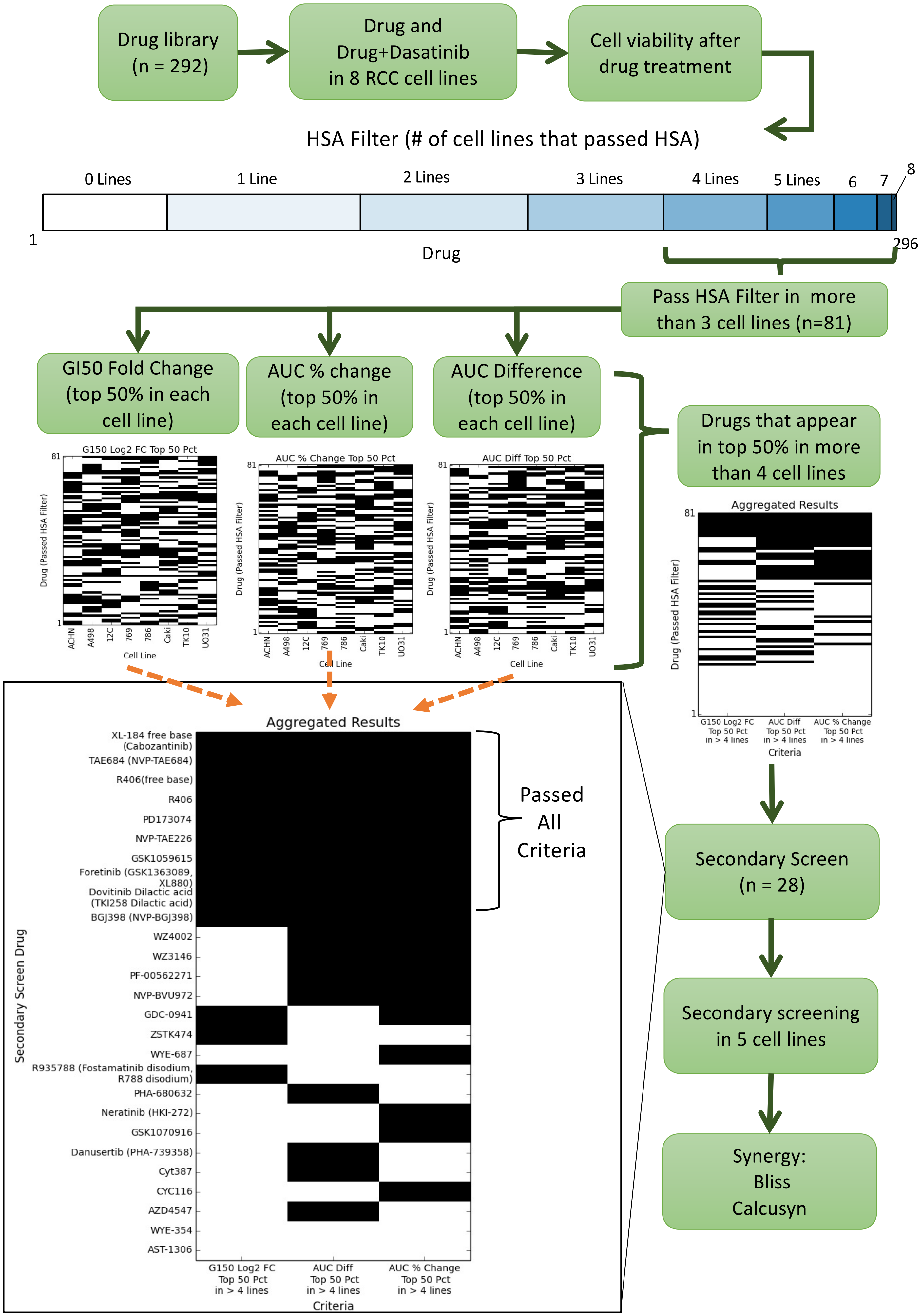

Drug library (n = 292)
Drug and Drug+Dasatinib in 8 RCC cell lines
Cell viability after drug treatment
HSA Filter (# of cell lines that passed HSA)
0 Lines
1 Line
2 Lines
3 Lines
4 Lines
5 Lines
6
7
8
1
296
Drug
Pass HSA Filter in more than 3 cell lines (n=81)
GI50 Fold Change (top 50% in each cell line)
AUC % change (top 50% in each cell line)
AUC Difference (top 50% in each cell line)
G150 Log2 FC Top 50 Pct
AUC % Change Top 50 Pct
AUC Diff Top 50 Pct
Drug (Passed HSA Filter)
Cell Line
ACHN
A498
12C
769
786
Caki
TK10
UO31
Drugs that appear in top 50% in more than 4 cell lines
Aggregated Results
G150 Log2 FC Top 50 Pct in > 4 lines
AUC Diff Top 50 Pct in > 4 lines
AUC % Change Top 50 Pct in > 4 lines
Criteria
Secondary Screen Drug
XL-184 free base (Cabozantinib)
TAE684 (NVP-TAE684)
R406(free base)
R406
PD173074
NVP-TAE226
GSK1059615
Foretinib (GSK1363089, XL880)
Dovitinib Dilactic acid (TKI258 Dilactic acid)
BGJ398 (NVP-BGJ398)
WZ4002
WZ3146
PF-00562271
NVP-BVU972
GDC-0941
ZSTK474
WYE-687
R935788 (Fostamatinib disodium, R788 disodium)
PHA-680632
Neratinib (HKI-272)
GSK1070916
Danusertib (PHA-739358)
Cyt387
CYC116
AZD4547
WYE-354
AST-1306
Passed All Criteria
Secondary Screen (n = 28)
Secondary screening in 5 cell lines
Synergy: Bliss Calcusyn

Supplemental Figure 1: Overview of pipeline for drug selection. (Related to Figure 1).

The library of 292 drugs were screened in eight cell lines and retained cell viability. Eighty-one drugs passed the "Highest Single Agent" (HSA) filter in four or more lines (the HSA test requires that the combination have at least 10% greater inhibition than either dasatinib or the drug alone at the same dose, for at least three doses). Three measurements of these drugs were calculated for each cell line (G150 fold change, AUC percent change between drug alone and drug+dasatinib, and AUC difference between drug alone and drug+dasatinib). Each measurement results in a matrix where every (drug, cell line) pair is one if the measurement appears in the top 50% of measurements in the cell line and zero otherwise (three inset heatmaps). We then collapse each measurement matrix into a single column, where a drug's entry is one if more than half of the cell lines have the drug's measurement in the top 50%. These columns form the criteria matrix (*shown here for three criteria, shown for six criteria in Figure 1B)*. The drugs were shortlisted based on this screen as well as other considerations such as clinical utility, which were subjected to secondary screening in five cell lines.

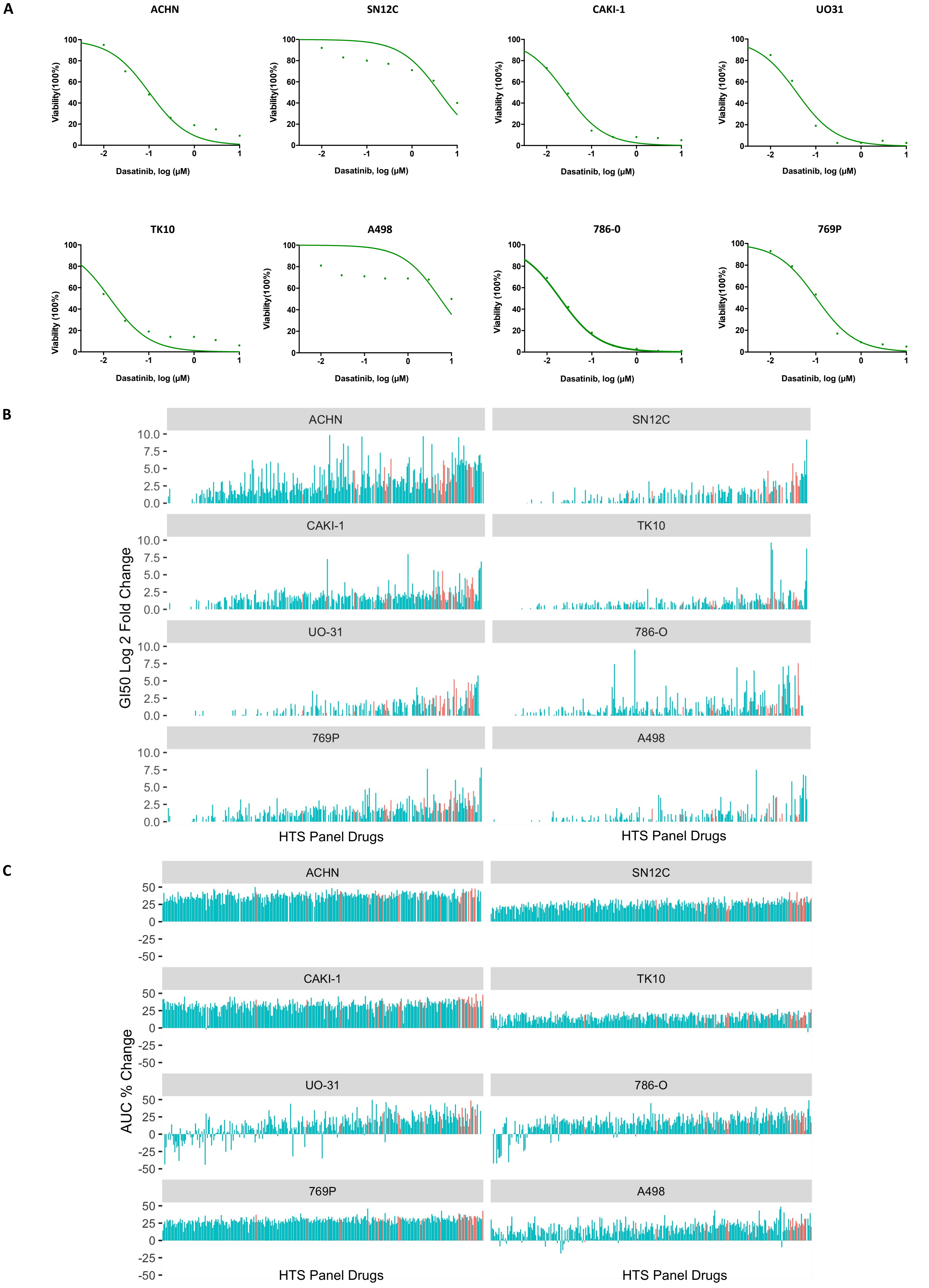
A
ACHN
SN12C
CAKI-1
UO31
TK10
A498
786-0
769P
Viability(100%)
Viability (100%)
Dasatinib, log (μM)
B
ACHN
SN12C
CAKI-1
TK10
UO-31
786-O
769P
A498
GI50 Log 2 Fold Change
HTS Panel Drugs
HTS Panel Drugs
C
ACHN
SN12C
CAKI-1
TK10
UO-31
786-O
769P
A498
AUC % Change
HTS Panel Drugs
HTS Panel Drugs

Supplemental Figure 2: Dose response curves of cell viability of human kidney cancer cells to dasatinib, and representation of the fold-change in GI50, and of the percentage change in AUC for each cell line. (Related to Figure 1).

A: Single agent dasatinib dose response curves: Cell viability was assessed by Cell Titer-Glo in ACHN, SN12C, CAKI-1, UO-31, TK10, A498, 786-0 and 769P human kidney cancer cells treated with escalating doses of dasatinib. The best-fit line represents the variable slope [log(inhibitor) *v* normalized response].

B: GI50 fold change: Screen data for all compounds tested against human RCC cells: ACHN, SN12C, CAKI-1, TK10, UO-31, 786-0, 769-P and A498. In each graph, the fold-change GI50 that resulted with the addition of dasatinib is represented on the y-axis (capped at 50 for uniformity). Each column on the x-axis represents one drug from the high throughput screen. Those drugs that were selected for the secondary screen are represented in red.

C: AUC % change: Screen data for all compounds tested against human RCC cells: ACHN, SN12C, CAKI-1, TK10, UO-31, 786-0, 769-P and A498. In each graph, the % change in AUC (area under dose response curve) that resulted with the addition of dasatinib is represented on the y-axis (capped at 50 for uniformity). Each column on the x-axis represents one drug from the high throughput screen Those drugs that were selected for the secondary screen are represented in red.

R406 (Growth Inhibition)

ACHN

Dasatinib (µM) \ R406 (µM)	0.0	0.0	0.6	2.1	3.7	5.3	7.6	10.0
0	0.00	0.02	0.14	0.15	0.49	0.49	0.31	0.36
0.01	0.04	0.06	0.27	0.47	0.80	0.76	0.53	0.57
0.0324	0.16	0.15	0.43	0.62	0.88	0.84	0.81	0.77
0.0552	0.25	0.25	0.53	0.81	0.89	0.88	0.87	0.88
0.08	0.30	0.36	0.60	0.86	0.87	0.89	0.89	0.88
0.096	0.38	0.38	0.67	0.84	0.90	0.90	0.88	0.90

PD173074 (Growth Inhibition)

Dasatinib (µM) \ PD173074 (µM)	0.0	0.0	0.1	0.3	0.5	0.7	1.1	1.6
0	0.00	0.00	0.05	0.02	0.00	0.02	0.07	0.28
0.01	0.04	0.08	0.06	0.08	0.14	0.18	0.27	0.43
0.0324	0.16	0.20	0.19	0.27	0.23	0.42	0.59	0.58
0.0552	0.25	0.28	0.20	0.39	0.45	0.47	0.65	0.78
0.08	0.30	0.37	0.37	0.47	0.48	0.53	0.70	0.70
0.096	0.38	0.35	0.49	0.59	0.61	0.67	0.59	0.75

SN12C

Dasatinib (µM)	0.0	0.1	0.2	0.5	0.8	1.1	1.8	2.5
0	0.00	-0.65	-0.01	-0.07	0.26	0.25	0.23	0.25
0.01	0.13	0.15	-0.19	0.37	0.48	0.38	0.54	0.92
0.0275	0.26	0.28	-0.13	0.73	0.72	0.45	0.90	0.98
0.045	0.35	0.32	0.48	0.80	0.86	0.89	0.99	0.99
0.0625	0.40	0.44	0.57	0.87	0.95	0.98	0.99	0.99
0.08	0.48	0.23	0.53	0.94	0.99	0.99	0.99	0.99

Dasatinib (µM)	0.0	0.1	0.9	3.0	5.2	7.4	8.7	10.0
0	0.00	0.21	0.19	0.25	0.51	0.64	0.71	0.69
0.04	0.28	0.43	0.47	0.62	0.77	0.82	0.86	0.85
0.13	0.33	0.58	0.61	0.70	0.82	0.85	0.87	0.88
0.22	0.33	0.63	0.63	0.71	0.80	0.86	0.88	0.89
0.31	0.36	0.63	0.65	0.76	0.83	0.88	0.87	0.90
0.4	0.38	0.67	0.70	0.76	0.82	0.87	0.88	0.91

CAKI-1

Dasatinib (µM)	0.0	0.1	0.2	0.5	0.8	1.1	1.8	2.5
0	0.00	-0.65	-0.01	-0.07	0.26	0.25	0.23	0.25
0.01	0.13	0.15	-0.19	0.37	0.48	0.38	0.54	0.92
0.0275	0.26	0.28	-0.13	0.73	0.72	0.45	0.90	0.98
0.045	0.35	0.32	0.48	0.80	0.86	0.89	0.99	0.99
0.0625	0.40	0.44	0.57	0.87	0.95	0.98	0.99	0.99
0.08	0.48	0.23	0.53	0.94	0.99	0.99	0.99	0.99

Dasatinib (µM)	0.0	0.0	0.1	0.2	0.4	0.5	0.9	1.2
0	0.00	0.01	0.14	0.32	0.42	0.44	0.45	0.66
0.01	0.13	-0.05	0.32	0.66	0.74	0.84	0.85	0.87
0.0275	0.26	0.33	0.40	0.92	0.90	0.89	0.95	0.98
0.045	0.35	0.35	0.55	0.96	0.95	0.99	0.98	0.99
0.0625	0.40	0.66	0.89	0.97	0.99	0.99	0.99	0.99
0.08	0.48	0.50	0.79	0.99	0.99	0.99	0.99	0.99

786-0

Dasatinib (µM)	0.0	0.1	0.2	0.6	1.0	1.4	2.3	3.2
0	0.00	0.08	0.10	0.10	0.28	0.28	0.65	0.77
0.01	0.17	0.22	0.25	0.40	0.56	0.71	0.81	0.83
0.0275	0.39	0.49	0.54	0.63	0.77	0.77	0.85	0.85
0.045	0.45	0.54	0.55	0.75	0.77	0.85	0.89	0.87
0.0625	0.52	0.63	0.67	0.80	0.83	0.87	0.90	0.92
0.08	0.54	0.48	0.56	0.72	0.78	0.79	0.81	0.81

Dasatinib (µM)	0.0	0.3	0.4	0.9	1.4	2.0	3.3	4.7
0	0.00	0.01	0.01	0.00	0.00	0.00	0.03	0.21
0.01	0.17	0.36	0.32	0.33	0.34	0.42	0.38	0.68
0.0275	0.39	0.51	0.56	0.53	0.52	0.56	0.63	0.76
0.045	0.45	0.61	0.50	0.56	0.56	0.64	0.70	0.80
0.0625	0.52	0.60	0.64	0.63	0.66	0.64	0.70	0.82
0.08	0.54	0.57	0.57	0.53	0.53	0.55	0.67	0.81

769-P

Dasatinib (µM)	0.0	0.4	0.8	1.4	2.1	4.7	7.4	10.0
0	0.00	0.11	0.06	0.12	0.15	0.52	0.64	0.71
0.02	0.26	0.38	0.39	0.50	0.63	0.84	0.89	0.90
0.065	0.54	0.59	0.68	0.72	0.82	0.91	0.92	0.92
0.11	0.68	0.76	0.73	0.80	0.84	0.90	0.92	0.92
0.155	0.77	0.83	0.82	0.80	0.87	0.91	0.92	0.92
0.2	0.78	0.75	0.81	0.82	0.87	0.92	0.92	0.92

Dasatinib (µM)	0.0	0.5	0.7	1.4	2.1	4.7	7.4	10.0
0	0.00	0.06	0.06	0.02	0.06	0.00	0.20	0.63
0.02	0.26	0.33	0.31	0.27	0.29	0.41	0.51	0.73
0.065	0.54	0.64	0.65	0.63	0.63	0.69	0.72	0.81
0.11	0.68	0.70	0.74	0.73	0.75	0.81	0.79	0.84
0.155	0.77	0.79	0.80	0.79	0.79	0.79	0.84	0.87
0.2	0.78	0.83	0.82	0.81	0.83	0.85	0.89	0.90

GSK1059615 (Growth Inhibition)

ACHN

Dasatinib (µM) \ GSK1059615 (µM)	0.0	0.0	0.1	0.4	0.7	1.0	1.6	2.2
0	0.00	0.00	0.09	0.08	0.14	0.27	0.45	0.56
0.01	0.04	0.06	0.10	0.20	0.23	0.36	0.60	0.66
0.0324	0.16	0.18	0.13	0.33	0.33	0.52	0.66	0.71
0.0552	0.25	0.30	0.27	0.46	0.57	0.56	0.66	0.74
0.08	0.30	0.34	0.40	0.56	0.55	0.61	0.75	0.80
0.096	0.38	0.34	0.42	0.52	0.60	0.72	0.76	0.80

GDC0941 (Growth Inhibition)

Dasatinib (µM) \ GDC0941 (µM)	0.0	0.0	0.1	0.2	0.4	0.6	1.0	1.4
0	0.00	0.00	0.06	0.19	0.37	0.42	0.51	0.52
0.01	0.04	0.13	0.19	0.47	0.60	0.66	0.68	0.70
0.0324	0.16	0.32	0.49	0.69	0.73	0.76	0.80	0.81
0.0552	0.25	0.43	0.52	0.77	0.80	0.83	0.84	0.84
0.08	0.30	0.49	0.61	0.81	0.83	0.84	0.84	0.85
0.096	0.38	0.53	0.66	0.81	0.83	0.85	0.86	0.86

SN12C

Dasatinib (µM)	0.0	0.1	0.2	0.4	0.7	1.0	1.7	2.3
0	0.00	0.00	0.04	0.10	0.22	0.30	0.39	0.50
0.04	0.28	0.30	0.28	0.33	0.43	0.50	0.58	0.64
0.13	0.33	0.34	0.35	0.46	0.52	0.59	0.64	0.69
0.22	0.33	0.32	0.36	0.49	0.53	0.62	0.68	0.72
0.31	0.36	0.34	0.37	0.47	0.54	0.61	0.68	0.72
0.4	0.38	0.40	0.40	0.48	0.56	0.58	0.71	0.72

Dasatinib (µM)	0.0	0.1	0.2	0.7	1.2	1.7	2.8	3.9
0	0.00	0.12	0.15	0.28	0.30	0.35	0.43	0.52
0.04	0.28	0.34	0.38	0.45	0.53	0.58	0.64	0.67
0.13	0.33	0.43	0.50	0.55	0.58	0.63	0.70	0.75
0.22	0.33	0.45	0.51	0.58	0.66	0.68	0.68	0.75
0.31	0.36	0.43	0.49	0.58	0.58	0.66	0.69	0.74
0.4	0.38	0.49	0.51	0.60	0.68	0.70	0.71	0.72

CAKI-1

Dasatinib (µM)	0.0	0.0	0.1	0.2	0.3	0.4	0.6	0.8
0	0.00	-0.14	-0.09	0.13	-0.05	-0.03	-0.19	0.25
0.01	0.13	0.14	0.31	0.25	0.31	0.19	0.26	0.37
0.0275	0.26	0.43	0.45	0.48	0.10	0.51	0.46	0.58
0.045	0.35	0.49	0.49	0.54	0.49	0.44	0.51	0.65
0.0625	0.40	0.44	0.41	0.56	0.30	0.51	0.64	0.61
0.08	0.48	0.52	0.62	0.60	0.61	0.52	0.64	0.77

Dasatinib (µM)	0.0	0.0	0.1	0.2	0.4	0.5	0.8	1.1
0	0.00	-0.42	-0.18	-0.22	-0.03	0.11	0.15	0.23
0.01	0.13	0.23	-0.07	0.28	0.02	0.28	0.14	0.40
0.0275	0.26	0.45	0.33	0.37	0.43	0.49	0.54	0.60
0.045	0.35	0.20	0.43	0.18	0.37	0.64	0.74	0.76
0.0625	0.40	0.48	0.58	0.65	0.63	0.68	0.85	0.78
0.08	0.48	0.51	0.53	0.56	0.71	0.76	0.84	0.76

786-0

Dasatinib (µM)	0.0	0.0	0.1	0.2	0.3	0.4	0.6	0.9
0	0.00	0.00	0.05	0.00	0.08	0.16	0.17	0.28
0.01	0.17	0.22	0.13	0.21	0.33	0.48	0.47	0.73
0.0275	0.39	0.48	0.57	0.57	0.58	0.63	0.73	0.79
0.045	0.45	0.48	0.56	0.60	0.73	0.70	0.79	0.89
0.0625	0.52	0.57	0.64	0.67	0.75	0.77	0.85	0.88
0.08	0.54	0.60	0.60	0.66	0.72	0.75	0.82	0.89

Dasatinib (µM)	0.0	0.1	0.2	0.6	1.0	1.4	2.2	3.1
0	0.00	0.00	0.00	0.05	0.37	0.52	0.57	0.46
0.01	0.17	0.15	0.32	0.55	0.65	0.68	0.61	0.66
0.0275	0.39	0.60	0.66	0.73	0.70	0.71	0.77	0.78
0.045	0.45	0.62	0.69	0.78	0.76	0.74	0.78	0.86
0.0625	0.52	0.64	0.77	0.82	0.74	0.78	0.83	0.86
0.08	0.54	0.61	0.72	0.79	0.60	0.78	0.82	0.79

769-P

Dasatinib (µM)	0.0	0.1	0.6	1.4	2.1	4.8	7.4	10.0
0	0.00	0.03	0.17	0.29	0.56	0.73	0.84	0.84
0.02	0.26	0.37	0.51	0.67	0.74	0.86	0.91	0.93
0.065	0.54	0.59	0.76	0.79	0.85	0.93	0.94	0.96
0.11	0.68	0.74	0.82	0.87	0.90	0.94	0.95	0.96
0.155	0.77	0.82	0.86	0.90	0.92	0.94	0.96	0.96
0.2	0.78	0.84	0.87	0.91	0.93	0.94	0.96	0.96

Dasatinib (µM)	0.0	0.1	0.1	0.6	1.0	1.4	2.3	3.1
0	0.00	0.05	0.03	0.12	0.25	0.26	0.51	0.57
0.02	0.26	0.31	0.31	0.51	0.59	0.65	0.68	0.73
0.065	0.54	0.53	0.58	0.68	0.79	0.77	0.83	0.81
0.11	0.68	0.69	0.76	0.82	0.86	0.86	0.88	0.88
0.155	0.77	0.76	0.78	0.87	0.86	0.87	0.91	0.91
0.2	0.78	0.76	0.77	0.85	0.88	0.89	0.90	0.91

Supplemental Figure 3: Representation of secondary screening dose matrix. (Related to Figure 2).

Secondary screening dose matrix of the top ranked synergistic combinations with dasatinib (Calcusyn): R406, PD173074, GSK105961 and GDC0941 in human RCC cells: ACHN, SN12C; CAKI-1, 786-0 and 769-P. Growth inhibition was assessed after 4 days to varying doses of the stated drugs and dasatinib. Percent inhibition at each dose of the drug is presented.

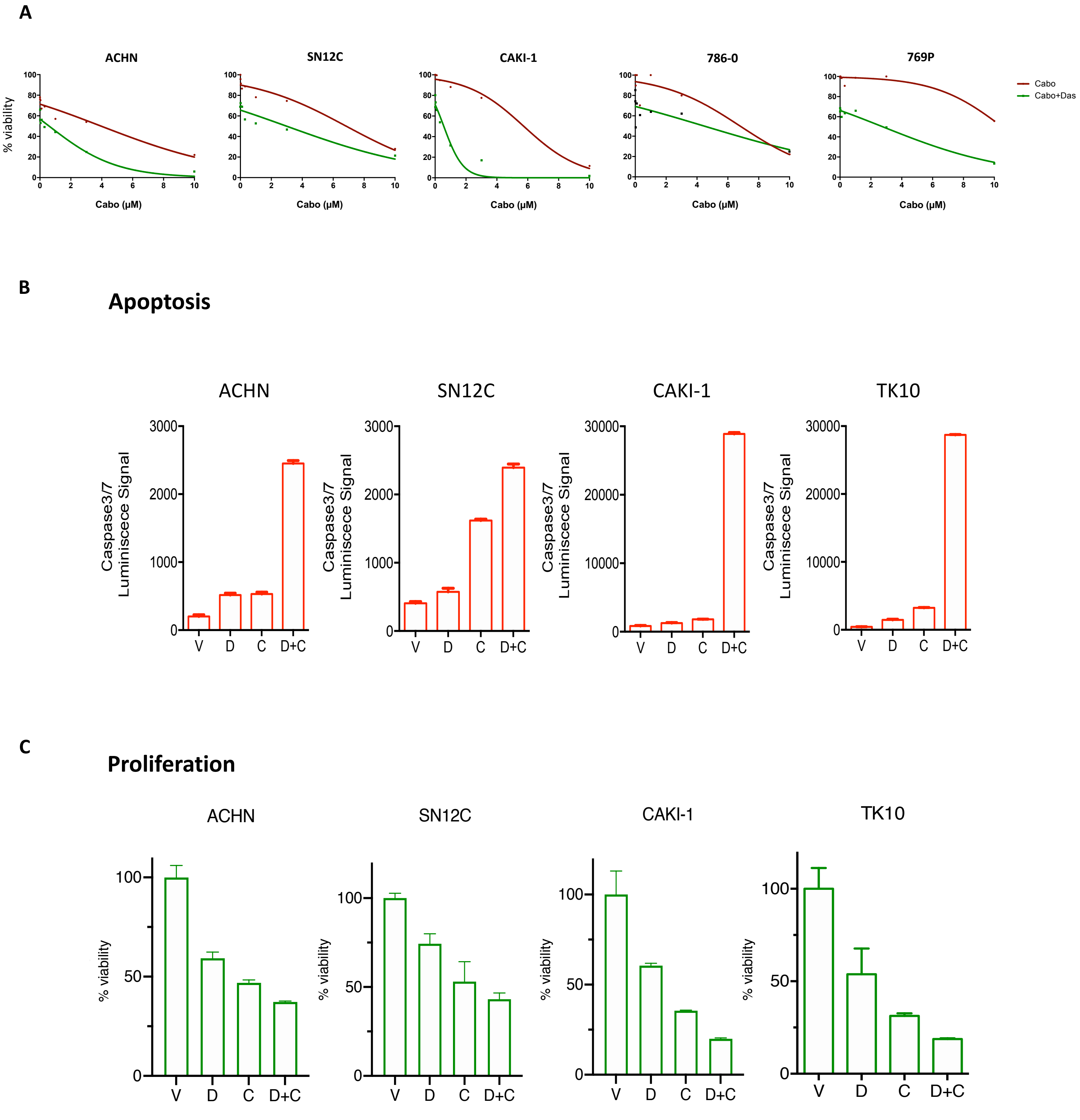

Supplemental Figure 4: Dose responses and effect of dasatinib and cabozantinib on apoptosis and proliferation. (Related to Figure 2)

A. Dose response to cabozantinib alone, and to the cabozantinib + dasatinib combination: Cell viability was assessed by CellTiter-Glo in ACHN, SN12C, CAKI-1, 786-0 and 769P human kidney cancer cells treated with escalating doses of cabozantinib alone (red line) or cabozantinib and a fixed dose of dasatinib at its IC_{25} for ACHN, SN12C, CAKI-1, 786-0, and 769P (green line). Represented as (inhibitor) *v.* normalized response-variable slope.

B. Apoptosis: Cell apoptosis measured by Caspase 3/7 compared to DMSO vehicle control (V) after 72hr-exposure of human RCC cells (ACHN, SN12C, CAKI-1, TK-10) to dasatinib (D: TK10: 10nM; ACHN, SN12C, CAKI-1: 50nM), cabozantinib (C: 10µM) or the combination (D+C); Representative results are shown. n = 2.

C. Proliferation: Cell viability measured by CellTiter Blue compared to DMSO vehicle control (V) after 72hr-exposure of human RCC cells (ACHN, SN12C, CAKI-1, TK-10) to dasatinib (D: TK10: 10nM; ACHN, SN12C, CAKI-1: 50nM), cabozantinib (C: 10µM) or the combination (D+C); Representative results are shown. n = 2.

A

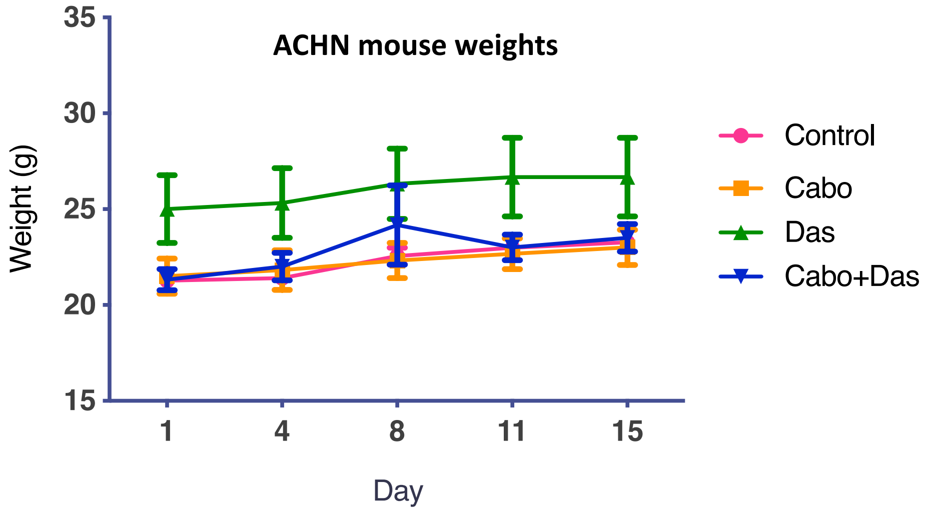

B

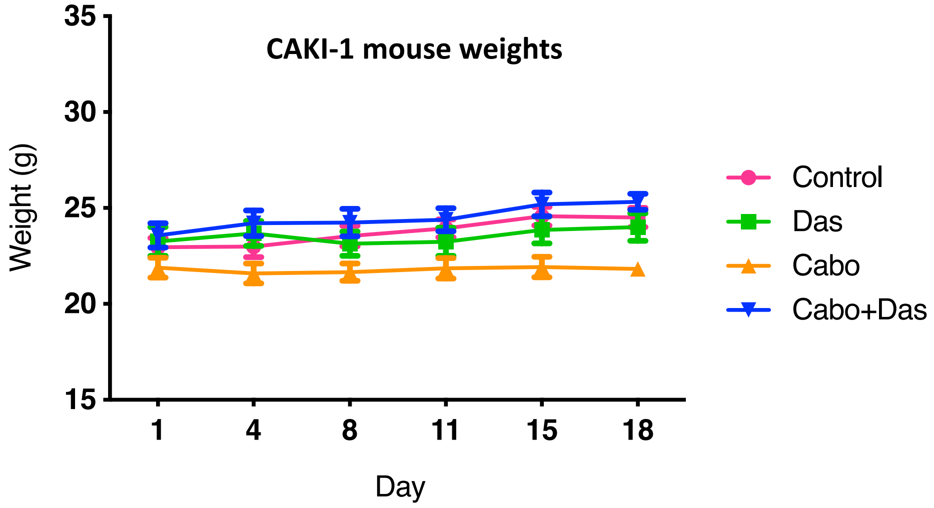

Supplemental Figure 5: Tolerability of dasatinib, cabozantinib and the combination in mouse xenograft models. (Related to Figure 3).

Body weights of mice bearing **(A)** ACHN, and, **(B)** CAKI-1 tumors as indicated.

Data are presented as mean ± SEM(n>8 per treatment group) ; *ns*: not significant; ACHN: control vs cabozantinib+dasatinib p=0.4778; CAKI-1: control vs cabozantinib+dasatinib, p=0.8833.

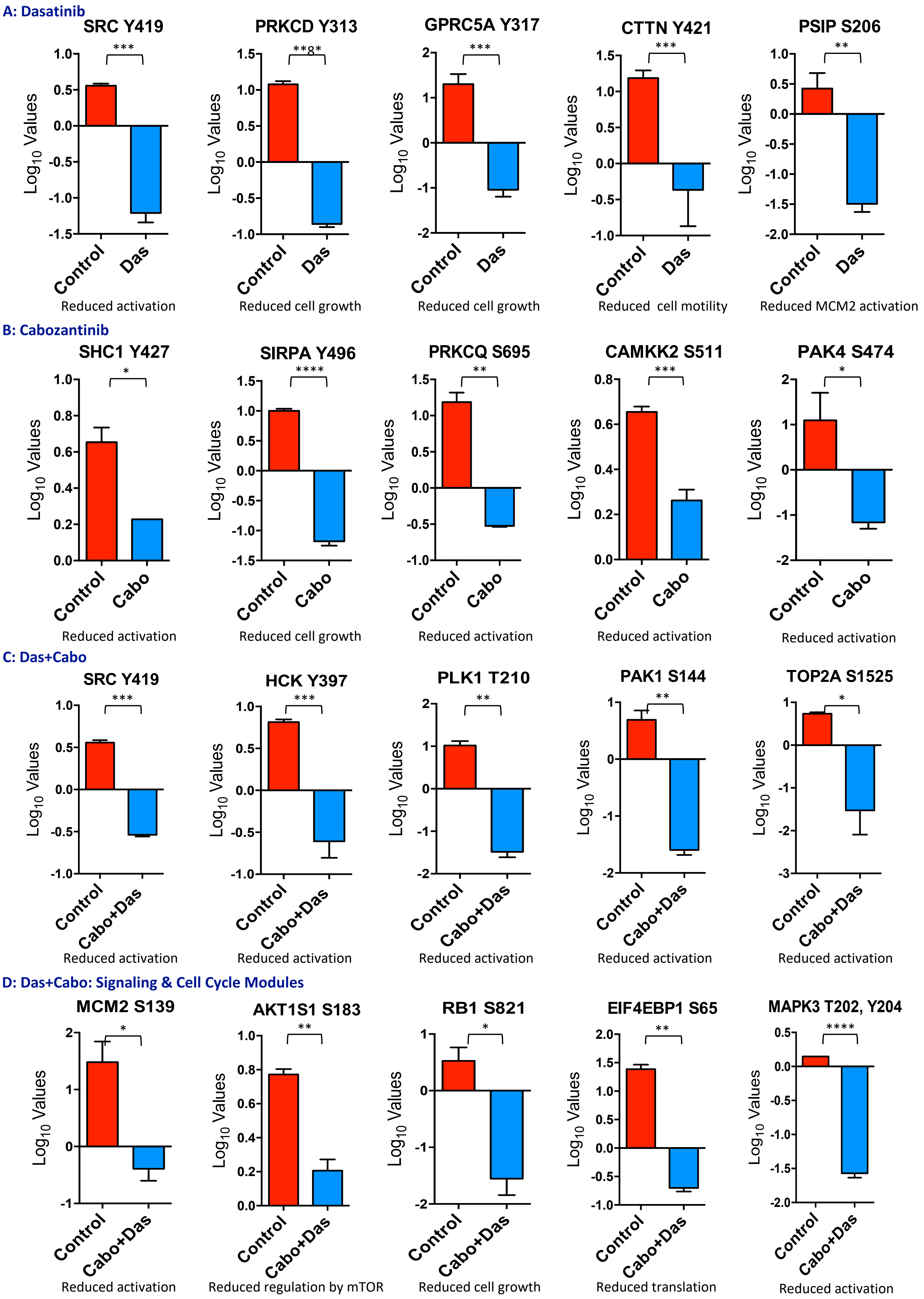
A: Dasatinib
SRC Y419
***
Log10 Values
Control
Das
Reduced activation
PRKCD Y313
**8*
Reduced cell growth
GPRC5A Y317
***
Reduced cell growth
CTTN Y421
***
Reduced cell motility
PSIP S206
**
Reduced MCM2 activation
B: Cabozantinib
SHC1 Y427
*
Cabo
Reduced activation
SIRPA Y496
****
Reduced cell growth
PRKCQ S695
**
Reduced activation
CAMKK2 S511
***
Reduced activation
PAK4 S474
*
Reduced activation
C: Das+Cabo
SRC Y419
***
Cabo+Das
Reduced activation
HCK Y397
***
Reduced activation
PLK1 T210
**
Reduced activation
PAK1 S144
**
Reduced activation
TOP2A S1525
*
Reduced activation
D: Das+Cabo: Signaling & Cell Cycle Modules
MCM2 S139
*
Reduced activation
AKT1S1 S183
**
Reduced regulation by mTOR
RB1 S821
*
Reduced cell growth
EIF4EBP1 S65
**
Reduced translation
MAPK3 T202, Y204
****
Reduced activation

Supplemental Figure 6: Effect of treatment on the phosphoproteome. (Related to Figure 4).

Evaluation of a select number of kinases and phosphoproteins from networks identified by KSEA: **A:** dasatinib, **B:** cabozantinib and, **C, D:** dasatinib-cabozantinib combination treated cells. Phosphoserine and phosphothreonine (pST) peptides and phosphotyrosine (pY) peptides identified from ACHN human NCCRCC cells with two technical replicates. The effect of treatment is stated below each graph, e.g., reduction in enzymatic activity. A t-test was performed to calculate significance. *p < 0.05, **p < 0.01, ***p < 0.001, ***p < 0.0001.

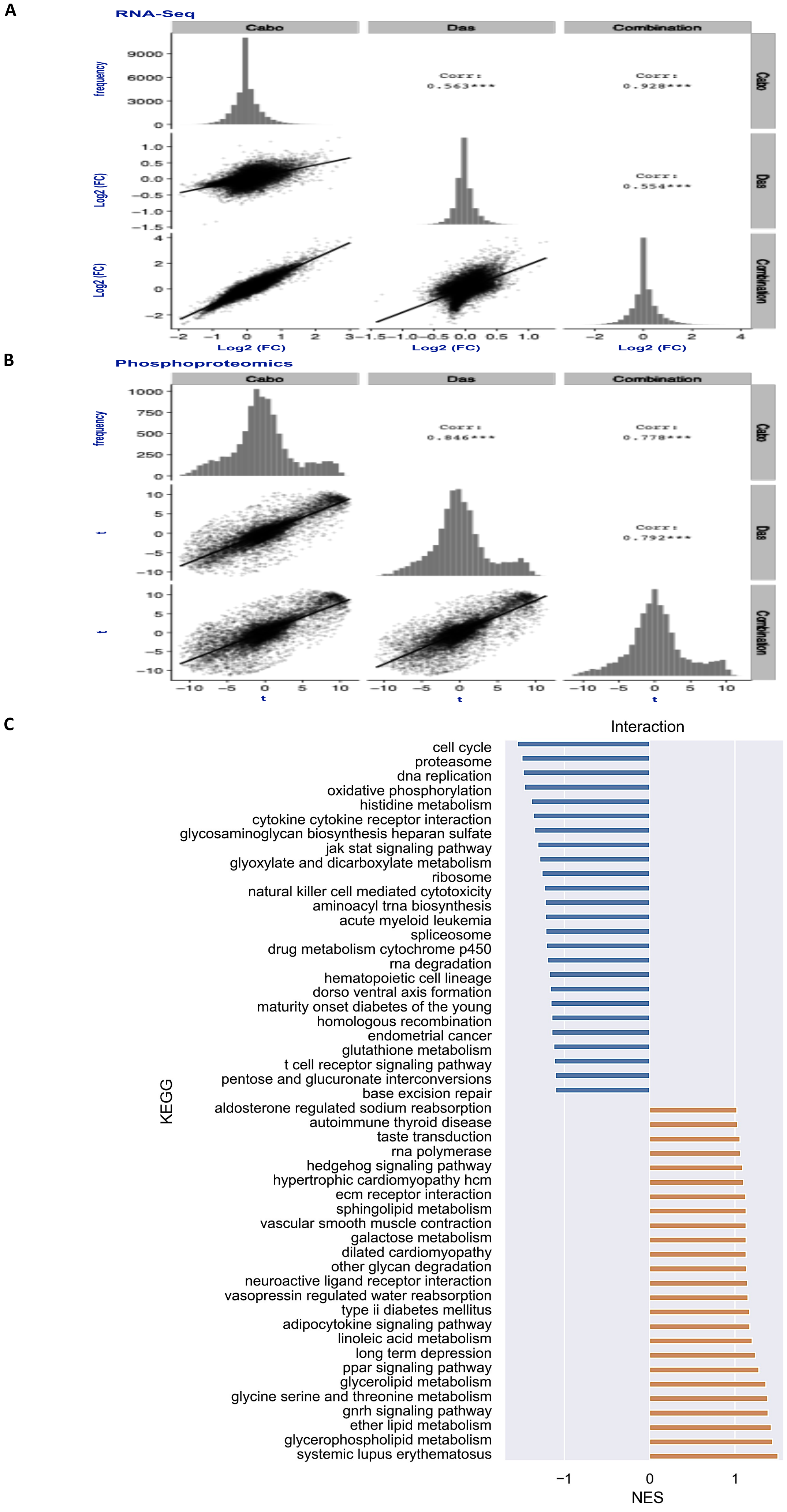
A
RNA-Seq
Cabo
Das
Combination
frequency
Log2 (FC)
Corr:
0.563***
Corr:
0.928***
Corr:
0.554***
Log2 (FC)
B
Phosphoproteomics
Cabo
Das
Combination
frequency
t
Corr:
0.846***
Corr:
0.778***
Corr:
0.792***
C
Interaction
KEGG
cell cycle
proteasome
dna replication
oxidative phosphorylation
histidine metabolism
cytokine cytokine receptor interaction
glycosaminoglycan biosynthesis heparan sulfate
jak stat signaling pathway
glyoxylate and dicarboxylate metabolism
ribosome
natural killer cell mediated cytotoxicity
aminoacyl trna biosynthesis
acute myeloid leukemia
spliceosome
drug metabolism cytochrome p450
rna degradation
hematopoietic cell lineage
dorso ventral axis formation
maturity onset diabetes of the young
homologous recombination
endometrial cancer
glutathione metabolism
t cell receptor signaling pathway
pentose and glucuronate interconversions
base excision repair
aldosterone regulated sodium reabsorption
autoimmune thyroid disease
taste transduction
rna polymerase
hedgehog signaling pathway
hypertrophic cardiomyopathy hcm
ecm receptor interaction
sphingolipid metabolism
vascular smooth muscle contraction
galactose metabolism
dilated cardiomyopathy
other glycan degradation
neuroactive ligand receptor interaction
vasopressin regulated water reabsorption
type ii diabetes mellitus
adipocytokine signaling pathway
linoleic acid metabolism
long term depression
ppar signaling pathway
glycerolipid metabolism
glycine serine and threonine metabolism
gnrh signaling pathway
ether lipid metabolism
glycerophospholipid metabolism
systemic lupus erythematosus
NES

Supplemental Figure 7: Scatterplots of gene expression and phosphoproteomics, and GSEA. (Related to Figure 5). Scatterplots of treatment-induced gene expression changes, in log_2 scale **(A),** and phosphoproteomic changes; **(B)**, as t statistics, with Pearson correlation R^2 values, for all pairwise combinations of treatments.

(C) GSEA analysis of the cabozantinib-dasatinib interactome: Gene set enrichment analysis (GSEA) was run on this ranked gene list (Kegg gene set). Normalized enrichment scores were used to generate the bar graph. Top 25 downregulated (blue) and upregulated (red) gene sets from the cabozantinib-dasatinib interactome are shown.

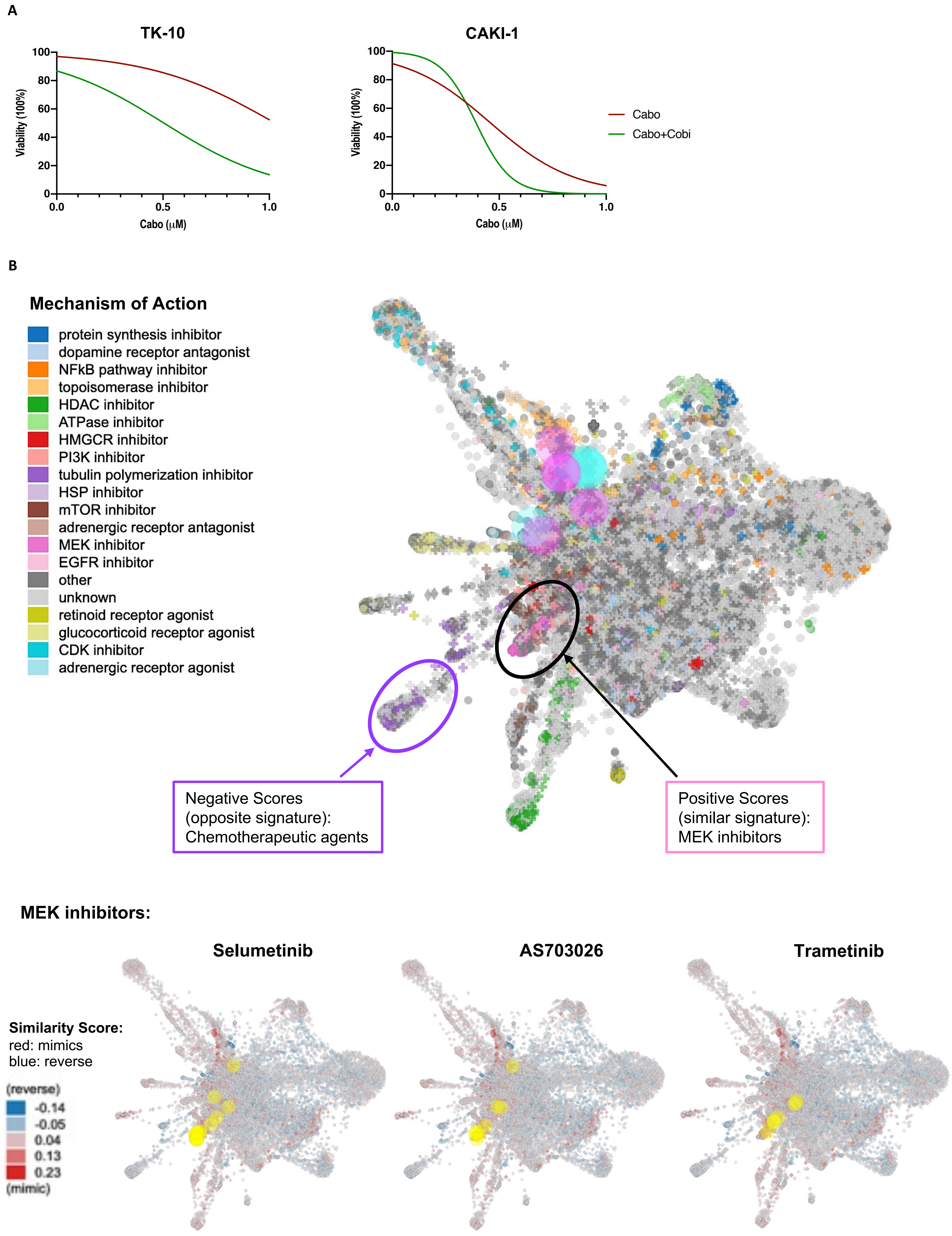
A
TK-10
CAKI-1
Viability (100%)
100
80
60
40
20
0
0.0
0.5
1.0
Cabo (μM)
Cabo
Cabo+Cobi
B
Mechanism of Action
protein synthesis inhibitor
dopamine receptor antagonist
NFkB pathway inhibitor
topoisomerase inhibitor
HDAC inhibitor
ATPase inhibitor
HMGCR inhibitor
PI3K inhibitor
tubulin polymerization inhibitor
HSP inhibitor
mTOR inhibitor
adrenergic receptor antagonist
MEK inhibitor
EGFR inhibitor
other
unknown
retinoid receptor agonist
glucocorticoid receptor agonist
CDK inhibitor
adrenergic receptor agonist
Negative Scores (opposite signature): Chemotherapeutic agents
Positive Scores (similar signature): MEK inhibitors
MEK inhibitors:
Selumetinib
AS703026
Trametinib
Similarity Score:
red: mimics
blue: reverse
(reverse)
-0.14
-0.05
0.04
0.13
0.23
(mimic)

Supplemental Figure 8: Examining cabozantinib-cobimetinib combinations, and extending the dasatinib-cabozantinib interaction signature to other cancer cells. **(Related to Figure 7).**

A: Dose response to cabozantinib alone, and to the cabozantinib + cobimetinib combination: Cell viability was assessed by CellTiter-Blue in TK-10 and CAKI-1 human NCCRCC cells treated with escalating doses of cabozantinib alone (red line) or cobimetinib (green line). Represented as (inhibitor) *v*. normalized response-variable slope.

B: L1000 Firework Display (L1000FWD) t-SNE visualization web tool was used to identify drug signatures and their corresponding mechanisms of action (MOA) that were similar to the dasatinib-cabozantinib interaction signature. Perturbation signatures were ranked based on the similarity score, revealing multiple MEK inhibitors, e.g., selumetinib, AS073026 and trametinib which are present in our screen, and highlighted in yellow on a L1000FWD t-SNE plot.
